# Blade-Coated All-Polymer Organic Solar Cells with 15% Efficiency Using Eco-Friendly Solvent Systems

**DOI:** 10.1021/acsami.5c12486

**Published:** 2025-10-08

**Authors:** Mohamed el Amine Kramdi, Aral Karahan, Lydia Abbassi, Takeshi Watanabe, Hidehiro Sekimoto, Olivier Margeat, Jörg Ackermann, Carmen M. Ruiz Herrero, Christine Videlot-Ackermann

**Affiliations:** † Aix Marseille Univ., CNRS UMR 7325, CINaM, Campus of Luminy, Case 913, Marseille 13288 Cedex 09, France; ‡ Aix Marseille Univ., Univ. de Toulon, UMR CNRS 7334, IM2NP, Technopôle de Château Gombert, 5 rue Enrico Fermi, Marseille 13453 Cedex 13, France; § Industrial Application Division, Japan Synchrotron Radiation Research Institute (JASRI), Sayo, Hyogo 679-5198, Japan; ∥ Department of Physical Science and Materials Engineering, Iwate University, Ueda, Morioka 020 8551, Japan

**Keywords:** all-polymer solar cells, blade coating technique, non-halogenated solvent, high efficiency, organic photovoltaics

## Abstract

Organic solar cells (OSCs) offer distinct advantages, such as solution processability, mechanical flexibility, and semitransparency. Recent advancements in polymerized small-molecule acceptors (PSMAs) enable high efficiencies in all-polymer solar cells (all-PSCs). As a promising candidate for next-generation organic photovoltaics, all-PSC technology holds a strong potential for large-scale commercialization, provided that device performance aligns with market demands. Critical factors influencing this transition include the development of environmentally friendly fabrication methods, as well as the optimization of active layer morphology and enhancement of charge transport layer quality. Currently, spin-coating is the predominant method for fabricating small-area OSCs, though it typically relies on toxic solvents, limiting its scalability and environmental compatibility. In this work, we report, for the first time, the scalable fabrication of PM6:PY-IT devices using the green solvent *o*-xylene and doctor-blade coating at ambient temperature in air. The resulting devices, fabricated in a conventional architecture incorporating PEDOT:PSS and PDINN interlayers, exhibited a short-circuit current density (*J*_sc_) of 22.54 mA/cm^2^, an open-circuit voltage (*V*_oc_) of 0.91 V, and a fill factor (FF) of 67.2% yielding a power conversion efficiency (PCE) of 15%. Key ink formulation steps, including controlled heating, stirring, and vortexing, enabled the optimization of film morphology and crystallinity, as confirmed by atomic force microscopy (AFM), transmission electron microscopy (TEM), and grazing-incidence wide-angle X-ray scattering (GIWAXS). Charge transport properties were subsequently evaluated via the space-charge limited current (SCLC) method. The combination of ambient, low-energy processing, and scalable deposition techniques underscores the potential of this approach for sustainable and efficient manufacturing of all-PSC devices.

## Graphical abstract

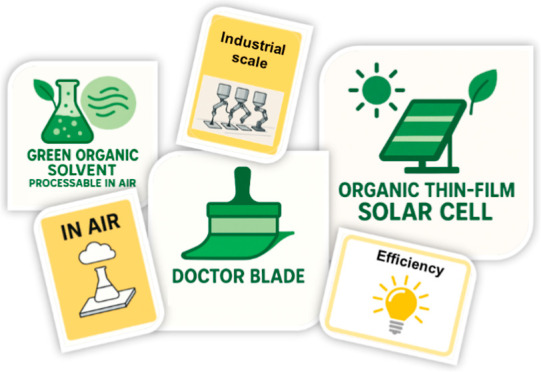

## 1 Introduction

Organic solar cells (OSCs) have been extensively studied because of their distinctive benefits, such as solution processability, mechanical flexibility, semitransparency, and potential for diverse outdoor applications.^1–3^ Thanks to the rapid progress in small molecular acceptor (SMA) design, numerous OSCs have demonstrated a power conversion efficiency (PCE) of around 20% based on SMAs with different chemical structures.^4–7^ Understanding the structure–property relationships derived from designing these SMAs creates new possibilities for the development of polymerized small-molecule acceptors (PSMAs). Meanwhile, all-polymer solar cells (all-PSCs), a subtype of OSCs, which use polymers as both donors and acceptors, are emerging as a promising class of devices. These cells are believed to exhibit excellent donor–acceptor compatibility and enhanced morphological stability, combined with excellent mechanical strength, positioning them as highly suitable candidates for large-scale commercialization. Recently, the emergence of PSMAs with the Y-series small molecular acceptors, used as primary building blocks, have demonstrated significant advancements in achieving higher PCEs for all-PSCs.^8–10^ Among these PSMAs, PY-IT has attracted considerable interest owing to its outstanding performance metrics in all-PSCs. In 2020, Luo et al. designed and synthesized PY-IT, a well-structured PSMA with a backbone similar to Y5 and linkers based on a thiophene unit.^11^

In the past few years, PY-IT, in combination with high-performance polymer donors such PM6 (see Figure 1 for chemical structures) have contributed to boosting the PCE of all-PSCs from 15.05% to 19.06% (see Table S1 and Figures S1 and S2 for more details).^12,13^ The high performance of this PM6:PY-IT binary blend has so far been achieved primarily with active layers deposited by spin-coating or layer-by-layer techniques, typically limited to small-area devices not exceeding 0.06 cm^2^. Another significant concern is that these high-performance devices are usually manufactured using highly toxic solvents, including chlorinated and aromatic solvents (chlorobenzene (CB), toluene) and chlorinated and nonaromatic solvent (chloroform (CF)), due to their excellent dissolving capability for highly conjugated structures (Figure S1). This approach is not suitable for large-scale production and has become a significant challenge hindering the mass production of organic photovoltaic (OPV) cells.

**Figure 1:**
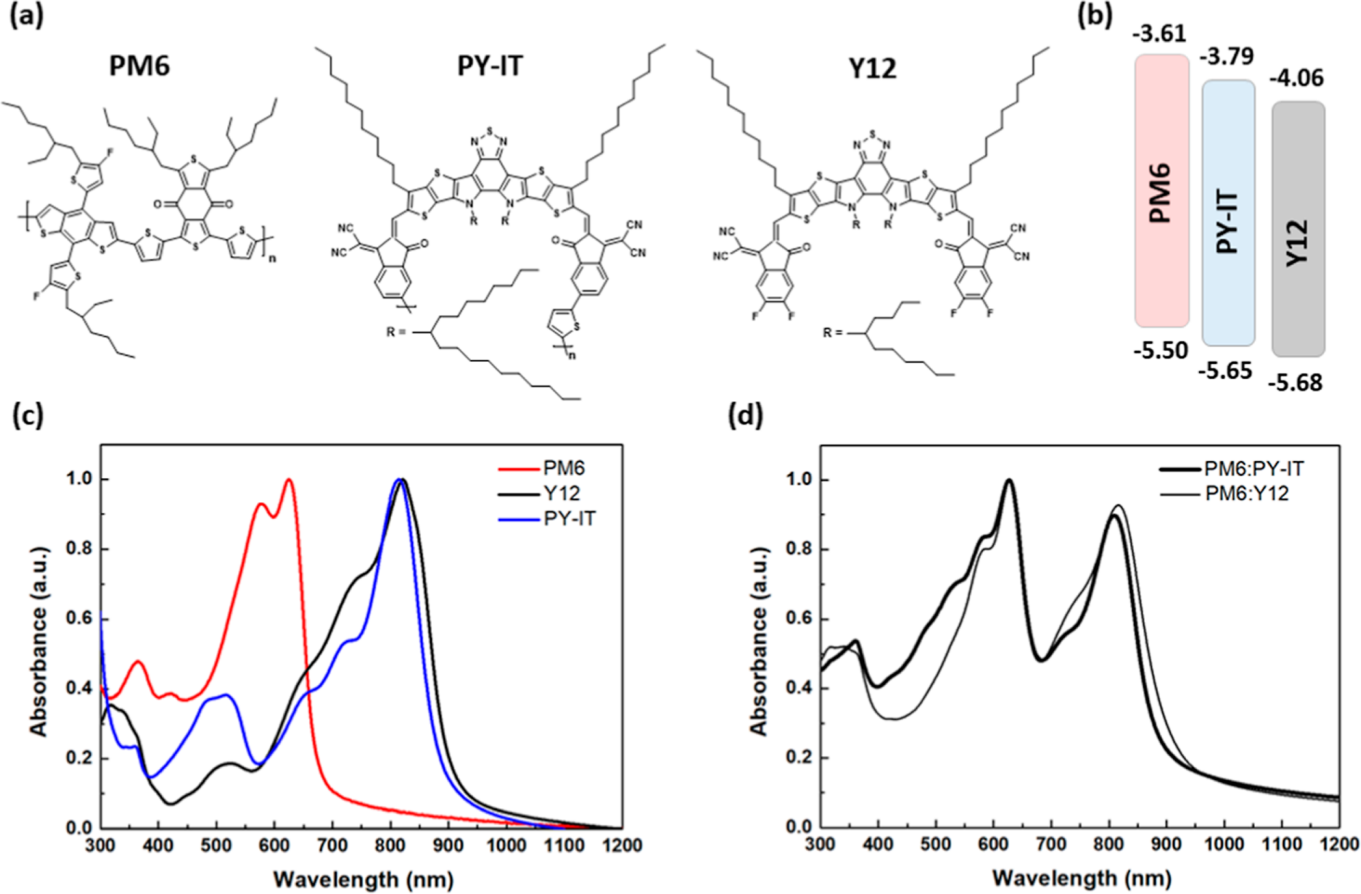
Chemical structure (a) and energy levels (b) of PM6, PY-IT, and Y12. (c) Absorption spectra of neat materials in thin films. (d) Absorption spectra of PM6:PY-IT (ratio 1:1) and PM6:Y12 (ratio 1:1.2) blend thin films.

Considerable attention has been devoted to the use of green solvents in active layer processing due to their eco-friendly properties and potential for scalable production. Among them, *o*-xylene (*ortho*-xylene) is a nonhalogenated aromatic hydrocarbon with the formula C_6_H_4_(CH_3_)_2_. It is widely used as a solvent in various industries, including pharmaceuticals, printing, rubber, cleaning agents, and paints. *O*-Xylene is regarded as a promising solvent for enabling green-solvent-processable OPV devices.^14^ Using *o*-xylene in the PM6:PY-IT blends, Yang et al. demonstrated a PCE of 16.22%, surpassing the PCE values achieved with toluene or 1,2,4-trimethylbenzene (TMB)-based all-PSCs.^15^ The superior performance of *o*-xylene is believed to result from a well-formed face-on molecular packing, suitable crystallinity, and an optimal phase separation length scale. These factors contribute to efficient and balanced charge transport as well as sufficient charge generation, thereby enhancing both *J*_SC_ and FF. However, the PM6:PY-IT blend solution was heated to 100 °C and then spin-coated at 2000 rpm onto PEDOT:PSS films preheated to 95 °C. These elevated manufacturing temperatures can pose substantial limitations. Consequently, OPV material design should address both performance and processability considerations at low processing temperature in air with environmentally friendly solvent while also scaling up to larger surfaces.

Achieving the desired morphology in the bulk heterojunction (BHJ) layer of all-PSCs is increasingly challenging due to the complex intermixing of two closely intertwined polymer components. This mixing becomes even more challenging if one or two materials are poorly soluble in green solvents such as *o*-xylene. Regulating aggregation in solution is crucial for optimizing the performance of OPVs. Appropriate additives, as 1-chloronaphthalene (CN), have been introduced as a simple and convenient strategy as solubilizing agents that improve the dispersibility of polymers in solvents (see Table S1).^12^ These agents modify the interactions of the polymer chains, making them more soluble and easier to process. In thin films, the efficiency of all-PSCs can be further enhanced by optimizing phase separation for effective exciton dissociation while also improving the crystallization and molecular orientation in the active layer to ensure efficient hole and electron transport.

In addition, OPV devices must be processed not only using green solvents but also with fabrication methods that reduce environmental impact, highlighting their critical role in the advancement of eco-friendly and sustainable energy solutions. Doctor-blade coating is a scalable, solution-based technique that can easily operate under ambient conditions and enables lower-temperature processing.^16,17^ A major challenge lies in maintaining PCEs when transitioning from the widely used spin-coating technique to scalable printing methods such as doctor-blade coating, as OPV devices processed by these methods often exhibit significantly lower performance.

To date, no studies have reported the fabrication of solar cells based on the PM6:PY-IT blend dissolved in *o*-xylene and deposited via an air-processed doctor-blade coating. This novel approach combines the advantages of environmentally friendly solvent processing with scalable film deposition techniques, offering promising potential for cost-effective and sustainable organic photovoltaic device manufacturing. In this work, we explore, for the first time, the optoelectronic properties and device performance of PM6:PY-IT active layers prepared using *o*-xylene as a green solvent and a doctor-blade coating under ambient conditions. Additionally, PM6:Y12-based solar cells, as part of the cutting-edge research into OPVs employing a small-molecule acceptor, are fabricated and used as reference devices for comparison.^18–21^ We demonstrate that meticulous ink formulation, including critical steps such as heating with magnetic stirring and vortex mixing, is crucial for enhancing both the photovoltaic performance and the processability of highly efficient all-polymer OPV cells. The ambient-temperature deposition used by the doctor-blade technique significantly reduces energy consumption, highlighting the efficiency and sustainability of this technique. The resulting active layers were thoroughly analyzed using atomic force microscopy (AFM), transmission electron microscopy (TEM), and grazing-incidence wide-angle X-ray scattering (GIWAXS) to investigate their morphology and crystallinity. Additionally, the charge transport properties were evaluated through the space-charge-limited current (SCLC) method, providing comprehensive insights into the device performance. Notably, solar cells based on the PM6:PY-IT blend exhibit performance levels comparable to those of PM6:Y12 reference devices under identical conditions, using *o*-xylene solutions and doctor-blade-deposited layers in air.

## 2 Experimental Section

### 2.1 Materials

Glass-patterned ITO substrates (size 25 × 25 mm) with 15 Ω/sq resistance were purchased from Lumtec, Taiwan. ZnO nanoparticle dispersion (N11 at 2.5%) was purchased from Avantama. Molybdenum­(VI) oxide powder (MoO_3_, purity 99.97%) and lithium fluoride (LiF, purity >99.99%) were purchased from Sigma-Aldrich (Merck), silver drops (Ag, purity 99.99%), and aluminum wire (Al, purity 99.999%) were from Kurt J. Lesker. *O*-Xylene (C_6_H_4_(CH_3_)_2_, anhydrous 97%) and 1,2,3,4-tetrahydronaphthalene (Tetralin) (analytical standard) were purchased from Sigma-Aldrich (Merck). PM6 (Mw ∼ 125–150 kDa, PDI ∼ 2.5) and Y12 were purchased from 1-Material and PY-IT from Solarmer (Mw = 18 kDa, purity >99%). PEDOT:PSS (CLEVIOS AI 4083) and PDINN (Mw = 674.83 g/mol, purity >98%) were purchased from Ossila. All commercial materials were used as received without purification and kept under a nitrogen atmosphere.

### 2.2 Ink Formulation and Preparation

Ink formulations based on PM6:Y12 and PM6:PY-IT blends were prepared in *o*-xylene with 3.5% volume of Tetralin, using a donor/acceptor ratio of 1:1.2 and total concentration of 20 mg/mL for PM6:Y12 and 1:1 with 15 mg/mL for PM6:PY-IT. The solutions were stirred at 65 °C overnight for a minimum of 15 h (step 1 in Figure 2). In the second step (step 2 in Figure 2), a vortex technique was applied for 30 s at room temperature. This vortexing procedure was repeated three times at 10 min intervals. During each interval, the solution was returned to the stirring plate without heating (step 3 in Figure 2). At the end of step 3, the inks were allowed to cool to ambient temperature before deposition. Additionally, as shown in Table S1 and Figure S2, and in line with the literature on PM6:PY-IT-based solar cells, PM6:PY-IT blends were also prepared by using an ink formulation with a 1:1.2 weight ratio at a concentration of 20 mg/mL.

**Figure 2:**
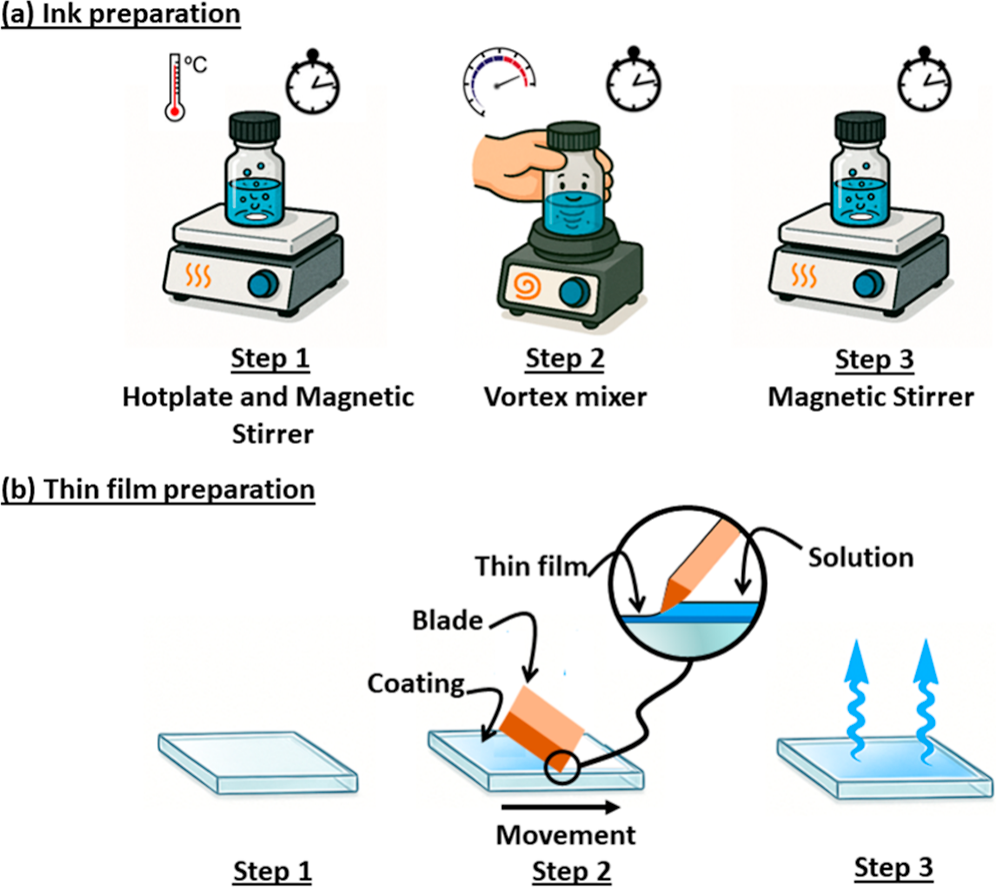
Schematic representations of ink preparation (a) and thin film preparation (b).

### 2.3 Solar Cell Fabrication

OPVs were fabricated in the conventional configuration of the ITO/PEDOT:PSS/active layer/PDINN/Ag. ITO substrates were thoroughly cleaned with a cloth dampened in isopropanol, followed by 30 min of sonication in isopropanol and a UV ozone treatment at 80 °C for 15 min. A thin layer of PEDOT:PSS was spin-coated dynamically under a fume hood at ambient conditions on the precleaned ITO glass substrate at a speed at 5000 rpm for 1 min to a thickness of 15–20 nm and transferred to the glovebox for annealing on a hot plate at 120 °C for 10 min. The active layer was then deposited by blade coating under ambient conditions. The HTL (3 nm of MoOx) and upper electrode (100 nm of Ag) were thermally evaporated inside the glovebox under a pressure of 2 × 10^–6^ mbar, using a designated shadow mask to achieve precise patterning (0.09 cm^2^ and 0.25 cm^2^).

### 2.4 Blade-Coating Process

The PM6:Y12 and PM6:PY-IT active layers were statically blade-coated in air and at ambient temperature with different sweep speeds. For the OPV-specific layers, sweep speeds of 1.7–1.9 mm/s were used, resulting in active layer thicknesses of ∼100 nm, respectively. The gap was fixed at 60 μm, and an ink volume of 30 μL was used for all formulations. Active layer films were annealed in air just after deposition at 100 °C for 5 min. After the active layers were deposited, the samples were transferred to the glovebox for thermal evaporation of the upper interfacial layer and electrode. For the SCLC-specific devices, sweep speeds ranging from 1.7 to 3 mm/s were employed, producing films with thicknesses varying between 71 and 485 nm. The gap was fixed at 60 μm, and an ink volume of 30 μL was used for all formulations. Active layer films were annealed in air just after deposition at 100 °C for 5 min.

### 2.5 Solar Cell Characterization

The current density–voltage (*J*–*V*) characteristics of the solar cells were measured inside the glovebox using a Keithley 238 Source Measure Unit and a Newport class AAA 1.5 Global solar simulator (Oriel Sol3ATM model n° 94043A) with an irradiation intensity of 100 mW/cm^2^. The light intensity was determined with a Si reference cell (Newport Company, Oriel n° 94043A) calibrated by the National Renewable Energy Laboratory (NREL). We present the performance of the best devices, whereas average PCEs were obtained with a standard deviation analysis calculated using 12 and 6 independent devices for 0.09 and 0.25 cm^2^, respectively.

External quantum efficiency (EQE) measurements were processed in a dark room in ambient conditions with a 150 W Xe arc lamp Apex illuminator (70525) light source collimated to a Cornerstone 260 1/4 m UV–vis Monochromator (74125) from Oriel Instruments for the light part and with a Keithley 238 Source Measure Unit for the electrical part. Devices were mounted on an optical table in a Faraday cage with an aperture to expose the samples to monochromatic light. The electrodes of the devices were electrically connected to the cage by spring loaded pogo pin connectors. The light beam was filtrated with 295 nm (10CGA-295) and 570 nm (10CGA-570) long-pass filters before reaching the devices.

### 2.6 Thin Film Characterizations

The absorbance of the active layer films was measured by a UV–vis–near-infrared Spectrophotometer Cary 5000. Film thicknesses were measured by a stylus profilometer (Bruker DEKTAK XT) with 1 mg of force on the probing tip. The surface morphology of the blend layers was investigated by AFM at room temperature with a NTEGRA from NT-MIDT in semicontact mode using the silicon tips (MikroMash) with a theoretical resonant frequency (*f*_0_) of 150 kHz and a force constant (*k*) of 5.4 N m^–1^ and a Dimension Nexus form Bruker in peakforce tapping mode with a scanasyst-air (5 nm of diameter, *k* = 0.4 N m^–1^, *f*_0_ = 70 kHz). The surface morphology of the blend layers was further analyzed using TEM with JEM-2100F set to 200 kV from the JEOL Company. Images were captured by an Orius camera on a DigitalMicrograph (GATAN). The films were transferred onto a grid (300 nm mesh copper) after the PEDOT:PSS layer was dissolved by immersing the glass/PEDOT:PSS/active layer substrate in water.

Thin films were further analyzed with high-brightness synchrotron radiation at BL19B2 in a SPring-8 (Japan). GIWAXS (Grazing-Incidence Wide-Angle X-ray Scattering) measurements were performed using a high-sensitive 2D X-ray detector (PILATUS 300 K). The incident angle and wavelength of X-rays were 0.13° and 0.100 nm, respectively.

The crystal coherence length (CCL) values were extracted by the Scherrer equation

1 $$\tau =\frac{K\lambda }{\beta \;\cos\theta }$$

where τ is the ordered (crystalline) domains (which may be smaller than or equal to the grain size), here defined as CCL and *K* is a constant (dimensionless shape factor) close to unity. The shape factor is typically equal to 0.9, λ is the wavelength of the X-ray, β is the full width at half-maximum (fwhm) of the diffraction peak in radians after subtracting the instrumental line broadening, and θ is the Bragg angle. From an analytical standpoint, fwhm was used to determine the length scale over which ordered regions (e.g., crystallites) diffract/scatter coherently via the Scherrer equation.

### 2.7 Fabrication and Measurements of Space Charge Limited Current Devices

Space Charge Limited Current (SCLC) devices require careful choice of the contacts to ensure sufficient injection of the desired carrier and effective blocking of the other carrier to work like single-carrier devices.^22–24^ Hole-only and electron-only devices consisted of ITO/PEDOT:PSS/active layer/MoO_3_/Ag and ITO/ZnO/active layer/LiF/Al, respectively. In SCLC-based devices, the mobility is measured vertically in the entire bulk of the active layer sandwiched between the two interfacial layers. Top metallic electrodes were thermally evaporated (MBRAUN evaporator inside the glovebox) at 2 × 10^–6^ mbar to a controlled thickness (5 nm of MoO_3_, 100 nm Au, 0.5 nm LiF, and 100 nm of Al) using a shadow mask that defines the device areas to 0.09 cm^2^ or 0.25 cm^2^ and allows a four-probe measurement. SCLC four-probe measurements were done in the same Faraday cage as for OPVs inside the glovebox. The thickness of thin films deposited by blade-coating was measured with a Veeco Dektak 150.

The measured dark current was fitted using the Murgatroyd expression

2 $$I=A\mu_{0}\frac{9}{8}\frac{V^{2}}{d^{3}}\varepsilon \varepsilon_{0}\;\exp\left(0.891\gamma \sqrt{\frac{V}{d}}\right)$$

where *d* is the active layer thickness, *A* is the active device area, εε_0_ is the permittivity of the active layer (ε is assumed equal to 3.5 and ε_0_ is the permittivity of free space), and *V* is the voltage. μ_0_ and γ are the unknown parameters that will be adjusted to get a good fit, all other parameters are fixed.^25^ μ_0_ is the mobility at low electric fields, and γ is a parameter that represents the field dependence of mobility. Measurements and analysis of *I*–*V* curves were made following a specific previously reported protocol.^22–24^ Detailed work examples and the corresponding data fitting (Figures S11 and S12) are given in the Supporting Information.

## 3 Results and Discussion

### 3.1 Optical Properties

Figure 1a shows the molecular structures of polymer donor PM6, polymer acceptor PY-IT, and small-molecule acceptor Y12 used in this study. Figure 1b illustrates the energy levels of the three materials. The highest occupied molecular orbital (HOMO) and lowest unoccupied molecular orbital (LUMO) levels for PM6 are −5.50 eV and −3.61 eV, respectively.^7^ Pure PY-IT exhibits lower HOMO and LUMO levels of −5.65 eV and −3.79 eV, respectively.^7^ These levels are further reduced for SMA Y12, with HOMO and LUMO values of −4.06 eV and −5.68 eV, respectively.^19^ Due to the high energy level of PY-IT, both the LUMO and HOMO offsets in the PM6:PY-IT system are less than 0.2 eV. Figure 1c shows the normalized ultraviolet––visible (UV–vis) absorption spectra of neat materials in thin films. The absorption of PM6 primarily occurs in the 450–700 nm range, while Y12 absorbs mainly in the 600–950 nm range, with a maximum at 821.5 nm. As a polymerized Y derivative, PY-IT absorbs predominantly in the 600–950 nm range with a maximum at 814 nm, extending the absorption spectra to the near-infrared (NIR) region. From solutions to films, significantly red-shifted peaks are observed for Y12 and PY-IT, indicating intermolecular π–π packing in the solid state and ordered aggregation in the film. As shown in Figure S3, the main absorption peak of Y12 and PY-IT in diluted chloroform (CF) solutions was located at 733 nm for Y12 and 787 nm for PY-IT. PY-IT exhibits the smaller red shift with 27 nm versus 89 nm for Y12, which may be related to its weakest intermolecular π–π packing related to its chain length. By combination of the materials to PM6:Y12 and PM6:PY-IT, complementary absorption properties are observed in the blended films. Figure 1d displays the normalized thin-film spectra of the blend films dissolved in *o*-xylene, the solvent used to enable these layers to form the active layer in the solar cells. To eliminate aggregates in solution and consequently in the thin films, inks were optimized prior to deposition using the doctor-blade technique. Figure 2a illustrates the three steps applied to the blend solutions (see the experimental part for details). This procedure was applied to both blends to ensure a consistent comparison of thin-film properties and device performance. To ensure the optimized dissolution of the materials in *o*-xylene, a combination of heating on a hot plate, magnetic stirring, and vortex mixing was employed. It has been demonstrated that vortex mixers generate more intense overall shear stresses compared to magnetic stirrers, where shear is primarily localized around the stirring bar. This results in a more uniform dispersion throughout the entire solution volume.^26^ Additionally, it is confirmed that high-shear mixing devices, such as vortex mixers, provide highly efficient mixing in complex media, enabling rapid and homogeneous dispersion of components.^27^ In a mixing of the D and A phases for OPV, an approach based on tiny vortex effects are generated within the rotational flow to effectively disperse the organic materials in the solvents, ensuring thorough dissolution.^28^ As a direct result, the vortex mixing technique introduced here effectively reduces both the size and the number of aggregates visible to the naked eye, as further confirmed in a following section by TEM. Thin films of both blends were fabricated via the doctor-blade deposition technique (Figure 2b). The thin-film preparation process was carried out in three main steps. First, a thin layer of PEDOT:PSS was deposited by spin-coating onto precleaned glass substrates coated with indium tin oxide (ITO), serving as the anode. It is important to note that this step was not performed for absorption measurements, for which glass substrates were used instead. Second, the active layer solution was deposited using a doctor-blade coater under ambient conditions, with optimized parameters such as blade height, coating speed, and the solution concentration to ensure uniform film formation. Finally, the films were allowed to dry on a hot plate at 100 °C for 5 min to promote solvent evaporation and proper film formation. Despite the different acceptor-to-donor ratio (D/A), the resulting two blend films exhibit identical absorption profiles for the acceptor component, which remains almost unchanged within the 750–950 nm range. The all-polymer-based ink formulated with PM6:PY-IT shows slightly higher absorption in the 350–550 nm range, featuring well-defined spectral structures that are precisely matched to the material properties (Figure S4). These observations confirm that each material, in particular, PY-IT, dissolves correctly in *o*-xylene, resulting in a homogeneous, well-intermixed blend. Furthermore, these results indicate that PM6:PY-IT has the potential to achieve a favorable short-circuit current density (*J*_SC_) in devices, which could enhance the EQE within the corresponding absorption regions.^29^

### 3.2 Surface Analysis

It is evident that a favorable film morphology plays a crucial role in the efficient performance of BHJ OSCs. To analyze the surface morphology and nanostructure of both systems, we utilized high-resolution AFM. Figure 3 compares the AFM images of the PM6:Y12 and PM6PY-IT layers. AFM images of neat PM6, Y12, and PY-IT materials are shown in Figure S5. According to the height image, the root-mean-square (rms) roughness values are evaluated. The rms roughness values of the different materials and blends were assessed at different scan sizes (0.5 × 0.5 μm^2^ and 2 × 2 μm^2^). The AFM topography of PM6 typically reveals a smooth and uniform surface with well-defined rice-shaped nanometer-scale grain features. The rms roughness ranges from 1.08 nm at a scan area of 0.5 × 0.5 μm^2^ to 1.45 nm at a 2 × 2 μm^2^ scan, with a slight increase in roughness observed at larger scales, which remains negligible for the performance of upscaled organic photovoltaic cells. Y12 shows a notably lower rms, with values of 0.28 nm at the 2 × 2 μm^2^, indicating a smoother surface compared to PM6. As neat materials, PY-IT exhibits the highest roughness with an rms value of ∼1.84 nm. At larger scales, more pronounced surface features such as particles with angular facets become visible. Notably, these particles appear even larger in the PY-IT film deposited from a solution prepared without vortex mixing, which is also associated with a significantly higher rms value of 2.31 nm (Figure S6). This demonstrates that the vortex mixing step effectively improves the dissolution and dispersion of the PY-IT polymer chains in an *o*-xylene solution to form a more homogeneous and finely distributed morphology.

**Figure 3:**
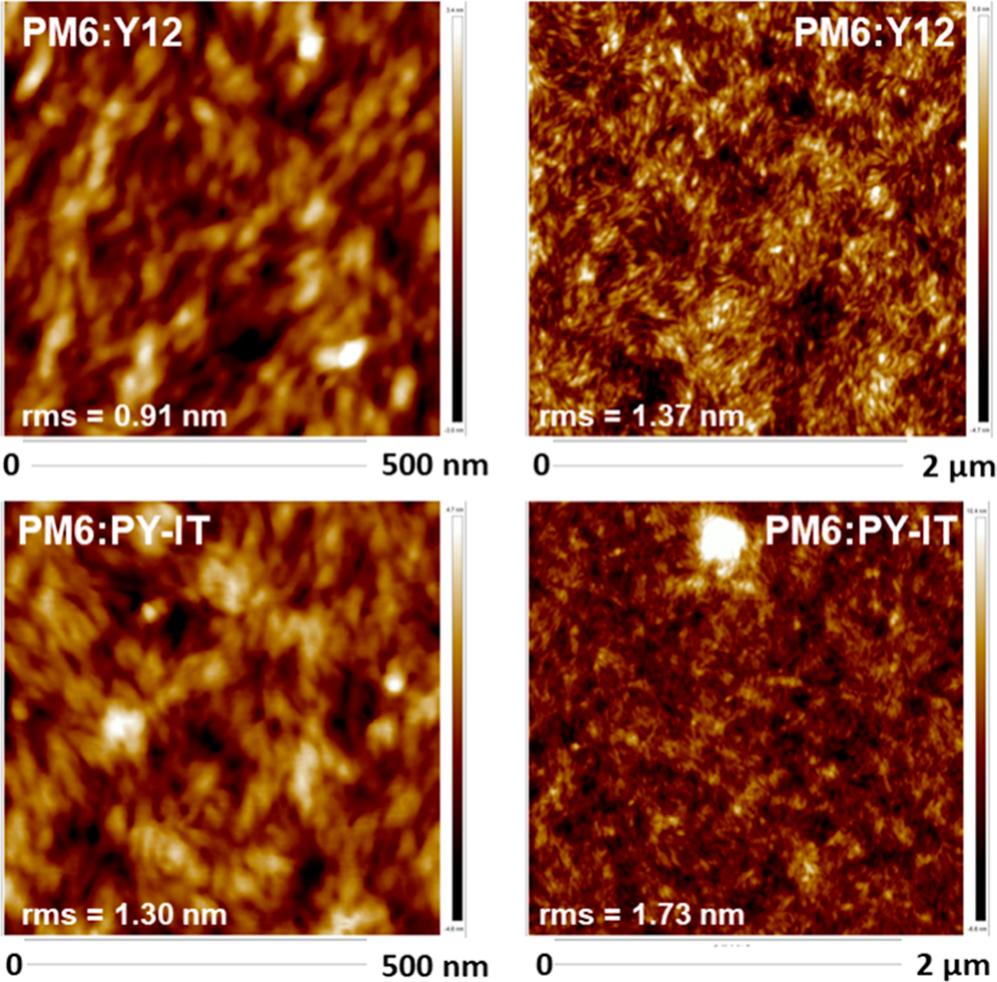
AFM images of PM6:Y12 and PM6:PY-IT blends. Both blends have been subjected to the vortexing procedure.

At the smallest scan size (0.5 × 0.5 μm^2^), the well-defined rice-shaped nanometer-scale grain features of PM6 remain predominant in blends. PM6:Y12 maintains relatively moderate roughness values, with 0.91 nm at 0.5 × 0.5 μm^2^ and 1.37 nm at 2 × 2 μm^2^, indicating uniformly distributed domains in a highly textured but continuous morphology (Figure 3). Despite no significant increase in roughness being observed for PM6:PY-IT blends (with 1.30 nm at 0.5 × 0.5 μm^2^ and 1.73 nm at 2 × 2 μm^2^), a prominent bright feature near the top center suggests the presence of a localized aggregate, a domain with significantly greater thickness (leading to an rms value of 2.17 nm when included in the calculation). Figure S6 compares the AFM images of PM6:Y12 and PM6:PY-IT layers with inks not subjected to the vortexing procedure. While the PM6:Y12 blend shows no significant change in its rms value, whether or not the vortex step is applied (1.37 vs 1.54 nm), the PM6:PY-IT blend exhibits a substantial increase, with the rms value reaching 6.16 nm at 2 × 2 μm^2^.

TEM was further used to investigate the nanostructural properties of the PM6:PY-IT blend films prepared by a blade coating (Figure S7). As shown in Figure S7a, PM6:PY-IT blend films prepared at room temperature without the vortex step exhibit numerous dark, circular particles aggregated into large clusters (120–240 nm). In contrast, the blend films prepared at the same temperature with the vortex step exhibit a substantially reduced number of aggregates (Figure S7b), with their size also significantly diminished (6–10 nm). In both thin films, a uniformly intermixed morphology is observed, apart from these aggregates, which is characteristic of nanoscale percolating networks. This further underscores the undeniable effectiveness of the vortex mixing step, particularly in all-polymer blends.

Finally, the fine morphology of both blends subjected to the vortexing procedure, characterized by small, well-defined features and a high surface coverage, is expected to promote the formation of an efficient donor–acceptor interface. This, in turn, should enhance charge carrier generation and transport within both the enriched donor and acceptor domains. Consequently, it will be crucial to assess whether this structural configuration significantly contributes to the PCE by comparing both inks subjected to or not to the vortexing procedure.

### 3.3 Bulk Analysis

GIWAXS measurements were performed to investigate the molecular stacking and crystallinity in the blend films. In GIWAXS, a very shallow incident angle is used, allowing the X-ray beam to penetrate deeply into the organic film before the reflected data are collected. For this reason, X-ray scattering techniques are widely employed to investigate the internal morphology of the films.^30^ In our study, GIWAXS measurements will therefore enable us to analyze the nanoscale percolating networks observed in TEM. Figure 4a shows the two-dimensional (2D) GIWAXS patterns of films based on PM6:Y12 and PM6:PY-IT. Figure 4b shows the one-dimensional (1D) line cuts of the in-plane (IP) and out-of-plane (OOP) directions of blend films (see Figure S8a for detailed extraction of both the OOP and IP line cuts for PM6:Y12). The film structure parameters measured from GIWAXS are listed in Table 1. In order to assign peaks to corresponding materials, GIWAXS measurements in pristine films have been done (Figure S9). PM6 deposited by doctor-blade in *o*-xylene exhibits the same crystalline structure as PM6 deposited by spin-coating in chlorobenzene (CB) and chloroform (CF).^23,31^ PM6 films consist of highly ordered crystallites as evidenced by an intense and narrow diffraction peak indexed as (100) at *q*_*z*_ = 0.30 Å^–1^ in the OOP profile. It is attributed to the edge-on lamellar stacking of PM6 with a lattice constant (*d*-spacing) of 2.09 nm. This feature in the GIWAXS spectra corresponds to well-defined, rice-shaped nanometer-scale grains observed by AFM. As a first observation, Y12 derivatives adopt a pronounced face-on orientation relative to the substrate with a sharp π–π stacking peak in the OOP profiles designated as the OOP (010) peak at ∼1.66–1.77 Å^–1^. This organization is further reinforced by the presence of the intense peak at ∼0.38 Å^–1^ in the IP. Regular molecular ordering in the IP direction is evident, as indicated by the presence of (100) and (200) lamellar peaks, highlighted in the zoomed-in image in Figure S9. This result is consistent with observations reported in the literature.^32^ PY-IT exhibits a distinct organizational structure, characterized by both a face-on orientation and a clearly defined edge-on arrangement, as (100) peaks appear in both IP and OOP profiles. Compared with the Y12 small molecules, PY-IT displays an isotropic organization, which also differs from the structure of the PM6 donor polymer.

**Figure 4:**
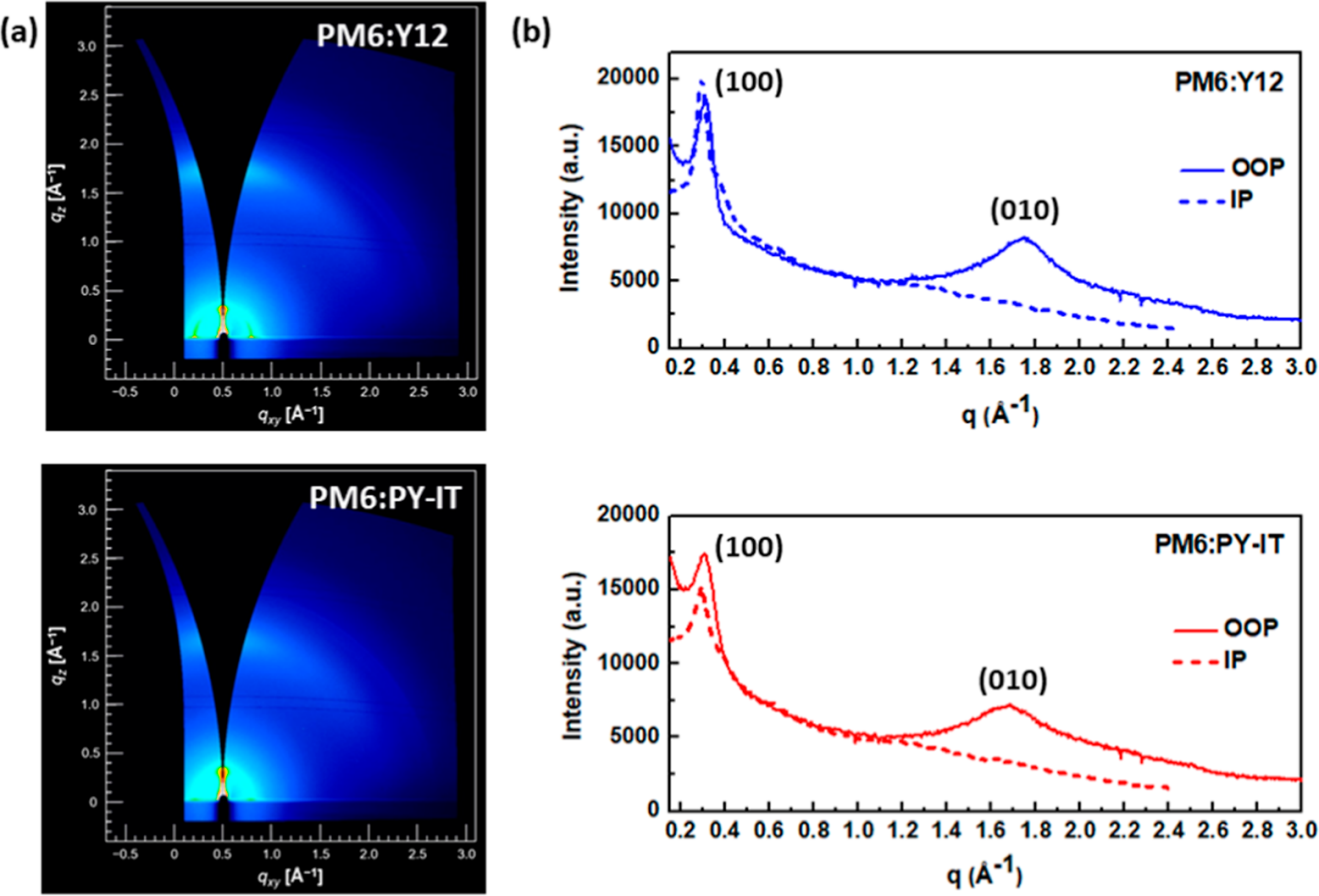
(a) 2D grazing-incidence X-ray diffraction patterns and (b) corresponding out-of-plane (OOP) and in-plane (IP) profiles of PM6:Y12 and PM6:PY-IT blends. Both blends have been subjected to the vortexing procedure.

**Table 1:** GIWAXS Measurements Data of the Scattering Vector (*q*) and π–π Stacking Distance (*d*) of Neat and Blend Films^a^

		peak (100)		peak (200)		peak (010)		
direction	sample	*q* (Å^–1^)	*d*_100_ (Å)	*q* (Å^–1^)	*d*_200_ (Å)	*q* (Å^–1^)	*d*_010_ (Å)	CCL (nm)
IP	PM6					1.66	3.78	
	Y12	0.28	22.2	0.38	16.5			
	PY-IT	0.30	20.9	0.38	16.5			
	PM6:Y12	0.29	21.6					
	PM6:PY-IT	0.29	21.6					
OOP	PM6	0.30	20.9					
	Y12					1.77	3.54	
	PY-IT	0.31	20.5			1.69	3.71	
	PM6:Y12	0.31	20.2			1.74	3.60	3.21
	PM6:PY-IT	0.30	20.9			1.69	3.73	2.57

a Crystal coherence length (CCL) of blend films are calculated in the OOP direction.

All blend films exhibit (100) peaks at ∼0.29–0.31 Å along both IP and OOP directions, along with a distinct (010) peak in the OOP direction. The distinct appearance of these three peaks clearly indicates an organization that reflects the interplay between the donor polymer chains PM6 and the acceptor molecules Y12 and PY-IT, with both blends showing remarkable similarity. While the OOP peak at 0.30–0.31 Å primarily arises from the lamellar stacking of PM6 polymer chains, the OOP peak at 1.69–1.74 Å highlights the face-on orientation of Y12 or PY-IT. These latter peaks correspond to the π–π stacking diffraction peaks with π–π stacking distances of 3.60 and 3.73 Å for the PM6:Y12 and PM6:PY-IT blends, respectively. The slightly shorter π–π stacking distance in the PM6:Y12 blend is attributed to the smaller π–π stacking distance (3.54 Å) of Y12, resulting from its simpler molecular structure. The CCL calculated by eq 1 in the OOP direction decreases for the all-polymer blend, from 3.21 nm in the PM6:Y12 blend to 2.57 nm in the PM6:PY-IT blend. These structural characteristics promote the formation of denser and bigger crystallites in PM6:Y12 compared to PM6:PY-IT. Additionally, the well-formed face-on molecular packing in blends will be highly advantageous for efficient charge transport in the layered stack configuration of OPV devices.^15^

Notably, the strict similarity between the two patterns disappears when the blend solutions are not subjected to the vortex step, highlighting its crucial role in defining the structural features (Figure S10). In the PM6:Y12 blends, the (100) peaks remain bright and sharp, whereas a significant change occurs when PM6 is combined with the PY-IT polymer. The (100) IP peak becomes markedly less intense, indicating that the high crystallinity of PM6 is suppressed by the incorporation of PY-IT, alongside a barely noticeable (010) peak. This clearly highlights the critical role of ink preparation in achieving well-defined crystalline layers of the two polymers. Ultimately, the vortex mixing step plays a crucial role in promoting both effective dissolution and uniform distribution of the polymers, underscoring its importance in achieving a finely distributed morphology. Notably, GIWAXS analysis provides insight into the molecular stacking and crystallinity of the fine structure observed in AFM and TEM. It confirms that the two polymers interact and mix effectively during the vortex step, establishing an effective donor–acceptor interface. The fine mixing of the polymers will need to be quantified by charge transport measurements within the layers.

### 3.4 Transport Properties

The SCLC method is commonly used to investigate charge transport properties in organic semiconductor materials involved in OPVs. The device typically consists of a sandwiched structure, where the organic layer serves as the active layer between the two electrodes. The configurations ITO/PEDOT:PSS/blend/MoOx/Ag and ITO/ZnO/blend/LiF/Al have been implemented as hole- and electron-only devices, respectively. Blends were deposited using the doctor-blade coating method as typically applied in OPV cell fabrication. The SCLC experiment is performed by applying a DC voltage across the device and measuring the resulting current. The *I*–*V* characteristics are recorded over a range of voltages, starting from 0 to ±4 V where the current exhibits a distinct transition from ohmic conduction to space-charge-limited current. The *I*–*V* data obtained from the SCLC experiment can be analyzed using models such as Mott–Gurney’s Law, which relates the current to the voltage and the thickness of the active layer. Hole (μ_h_) and electron (μ_e_) mobility values are extracted from the *I*–*V* curves of hole-only devices and electron-only devices, respectively. *I*–*V* curves were fitted using eq 2 in the SCLC region considering a field dependence of the mobility. A complete worked example for both hole and electron (Figures S11 and S12) and the corresponding data fitting are provided in the Supporting Information. μ_h_ and μ_e_ values obtained for PM6:Y12 and PM6:PY-IT layers are summarized in Table 2 and Figure 5. Based on hole-only devices, the hole mobility decreases slightly when blending PM6 with PY-IT, dropping from 5.9 × 10^–4^ cm^2^/V s in the PM6:Y12 layer to 2.09 × 10^–4^ cm^2^/V s in the PM6:PY-IT layer. This reduction in hole mobility is directly attributed to the lower degree of crystallinity in the PM6 domains when blended with PY-IT. The bulky polymeric chains of PY-IT are likely to disrupt the molecular packing and crystalline ordering of PM6 more significantly than the smaller Y12 molecules, which interfere less with PM6’s self-organization. Similarly, the electron mobility is also affected due to the polymeric nature of PY-IT (μ_e_ = 1.29 × 10^–4^ cm^2^/V s for PM6:PY-IT vs μ_e_ = 3.16 × 10^–4^ cm^2^/V s for PM6:Y12). This slight reduction in charge transport efficiency could directly contribute to a lower *J*_sc_ for PM6:PY-IT, as the photogenerated electrons are less effectively extracted from the device. Nevertheless, a continuous and effective electron transport pathway is preserved through efficient percolation within the PY-IT phase.

**Table 2:** Hole (μ_h_) and Electron (μ_e_) Mobility Values Obtained by SCLC of PM6:Y12 and PM6:PY-IT Bulk Heterojunction Layers^a^

blends		hole-only devices	electron-only devices	
composition	ratio	μ_h_ (cm^2^/V s)	μ_e_ (cm^2^/V s)	μ_h_/μ_e_
PM6:Y12	1:1.2	5.90 × 10^–4^ (±1.75 × 10^–4^)	3.16 × 10^–4^ (±1.19 × 10^–4^)	1.86
PM6:PY-IT	1:1	2.09 × 10^–4^ (±4.214 × 10^–5^)	1.29 × 10^–4^ (±4.45 × 10^–5^)	1.62
	1:1.2	1.90 × 10^–4^ (±3.74 × 10^–4^)	8.75 × 10^–5^ (±3.63 × 10^–5^)	2.17

a Standard deviations are indicated in brackets, from measurements on 16–24 devices. Film thickness ranging from 71 to 485 nm.

**Figure 5:**
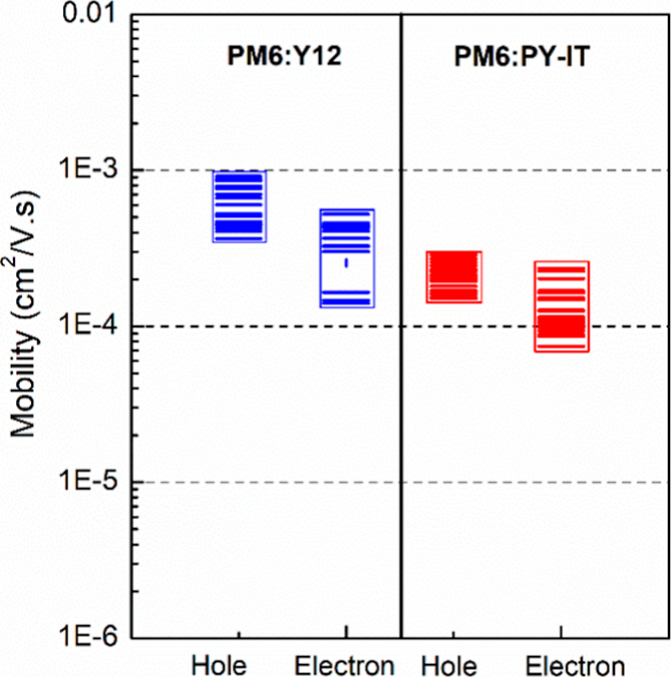
Distribution of hole and electron mobilities as a function of blend layer compositions: PM6:Y12 (1:1.2 ratio) and PM6:PY-IT (1:1 ratio).

Additionally, in both blends, the relatively well-balanced hole and electron mobilities are clearly maintained, establishing this balance as the key factor for efficient charge transport and overall device performance. Such balanced charge transport enables effective charge extraction, minimizes charge accumulation and recombination at the electrodes, and consequently can support a notable overall PCE. It is important to note that the mobility values for both holes and electrons are more affected for a 1:1.2 ratio, leading to a less favorable balance of these values (Figure S13 and Table 2). Consequently, as frequently reported in the literature (Figure S2), a 1:1 ratio between PM6 and PY-IT is established as the optimal composition.

### 3.5 Solar Cell Characterization

A series of OPVs were fabricated using the conventional configuration of ITO/PEDOT:PSS/active layer/PDINN/Ag. In this study, we investigate blends of PM6Y12 and PM6:PY-IT as SMA-based and all-polymer solar cells, respectively. The same batch of PM6 was used as the donor material in the fabrication of the devices. Both PM6:Y12 and PM6:PY-IT blends were optimized through the vortex mixing step. PDINN as a cathode interlayer material has demonstrated efficient contact with nonfullerene active layers, providing a stable electrode interface with a strong ability to reduce the work functions of metal cathodes. These properties make PDINN particularly suitable for use as a cathode interlayer in nonfullerene OSCs with air-stable metal cathodes, such as silver (Ag).^33,34^ In our experiment, we employed the doctor-blade coating method, which is a widely used technique for preparing printed organic layers in solar cells (Figure 2b). This noncontact printing technique involves depositing a solution between the blade and the substrate, forming a meniscus that drives the formation of the film. Film growth occurs naturally, governed by the adhesion between the solution’s bottom layer and the substrate. The resulting film thickness can be tuned by adjusting the solution concentration, the gap between the blade and substrate, and the coating speed. Precise control over these parameters is critical to achieving the desired film thickness and surface quality, while minimizing morphological defects, key factors for realizing high-performance printed OSCs. The thickness of the active layers was typically around 100 nm.

Figure 6a compares the current–density (*J*–*V*) curves of the best solar cells based on PM6:Y12 and PM6:PY-IT blends under an AM1.5G of 100 mW cm^–2^. Table 3 summarizes the photovoltaic parameters in terms of open-circuit voltage (*V*_oc_), charge current density (*J*_sc_), fill factor (FF), and power conversion efficiency (PCE) of the optimized solar cells, along with the average values and their standard deviation. Two cell areas were measured, 0.09 cm^2^ and 0.25 cm^2^. PM6:PY-IT blends deliver a *V*_oc_ of 0.91 V, which is ∼0.1 V higher than PM6:Y12. This behavior can be explained by the higher LUMO energy level of PY-IT with that of Y12, resulting in a larger energy offset with the HOMO of PM6.

**Figure 6:**
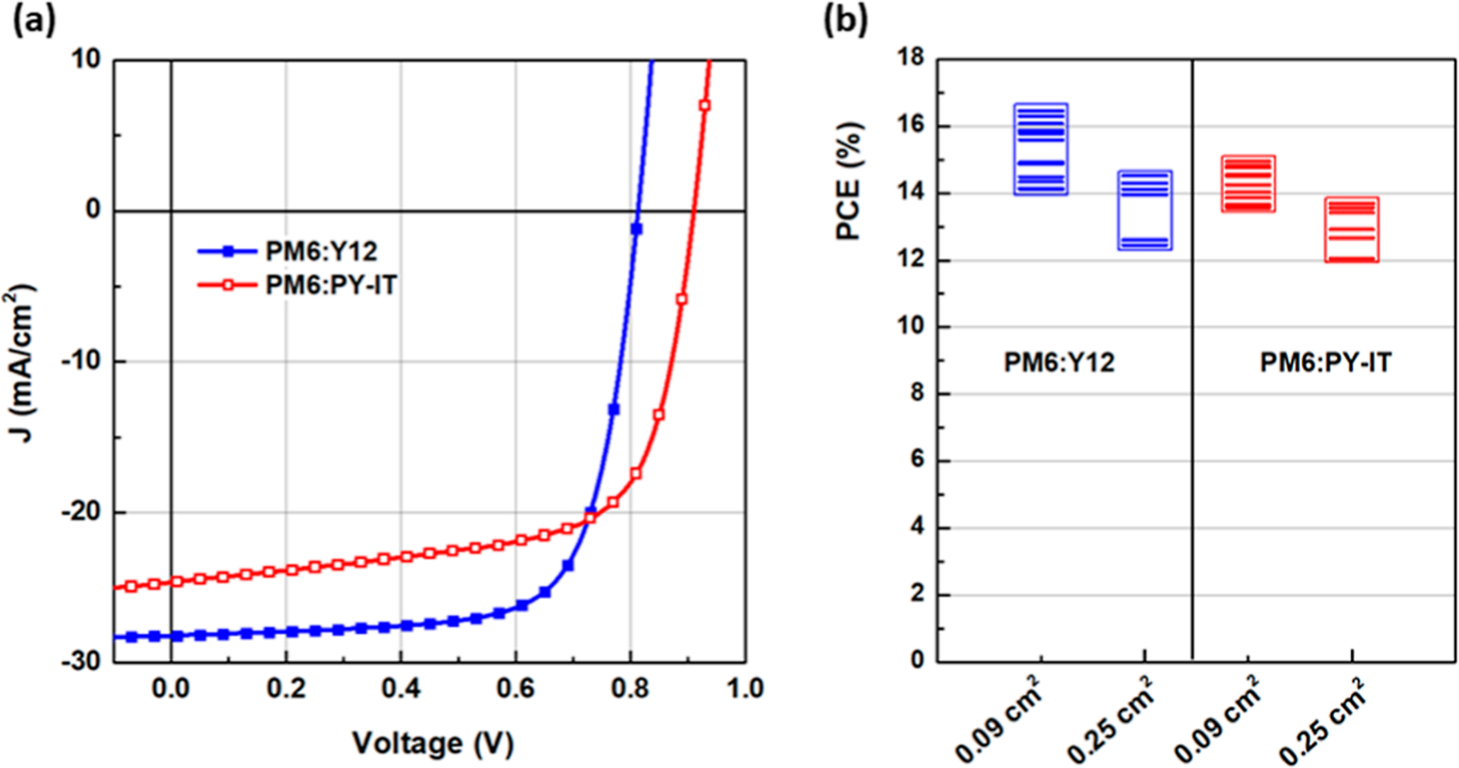
(a) *J*–*V* curves of OPVs based on PM6:Y12 and PM6:PY-IT blends. (b) PCE distribution as a function of the cell area.

**Table 3:** Photovoltaic Parameters of PM6:Y12 and PM6:PY-IT Blends as a Function of the Cell Area

	area (cm^2^)	*V*_oc_ (V)	*J*_sc_ (mA/cm^2^)	FF (%)	PCE (%)
PM6:Y12	0.09	0.813 (0.816 ± 0.004)	28.19 (26.52 ± 1.23)	71.80 (70.93 ± 2.20)	16.45^a^ (15.36 ± 0.79)^b^
	0.25	0.813 (0.817 ± 0.006)	25.25 (24.47 ± 0.98)	69.56 (68.22 ± 2.41)	14.29^a^ (13.65 ± 0.89)^b^
PM6:PY-IT	0.09	0.910 (0.907 ± 0.004)	24.54 (23.06 ± 0.92)	67.20 (68.11 ± 2.57)	15.00^a^ (14.24 ± 0.46)^b^
	0.25	0.910 (0.908 ± 0.008)	22.26 (21.60 ± 0.78)	67.52 (66.50 ± 1.98)	13.68^a^ (12.51 ± 1.52)^b^

a Values associated with the devices that showed the highest PCEs.

b Values in brackets are average based on 12 and 6 independent devices for 0.09 and 0.25 cm^2^, respectively.

The highest PCE of 16.45% is achieved with solar cells based on the PM6:Y12 blend, demonstrating exceptional performance in terms of *J*_sc_ and FF values. These efficiencies are equivalent to, or even surpass, the values reported in the literature for PM6:Y12 blend spin-coated devices treated with CF. Indeed, Baran et al. fabricated devices with a surface area of 0.1 cm^2^, achieving an efficiency of 15.5%, with a *J*_sc_ of 26.9 mA/cm^2^, a *V*_oc_ of 0.85 V, and an FF of 67.6%.^18^ An efficiency of 16.4% was achieved but with a surface area of ∼2.6 times smaller (0.038 cm^2^ vs 0.1 cm^2^).^19,20^ Additionally, our results are comparable to those reported for the PM6:Y12 blend when blade-coated in an inverted architecture (ITO/ZnO/PM6:Y12/MoO_3_/Ag). Notably, a maximum PCE of 15.6% was achieved using blade-coating with *o*-xylene as the solvent. However, no specific information regarding the device surface area is provided, leaving open the possibility of a relatively small surface, which strengthens the validity of our results. In our study, upscaling to a surface area of 0.25 cm^2^ achieved a maximum PCE of 14.29%, representing the largest surface area reported for solar cells based on blade-coated PM6:Y12 using a nonhalogenated solvent system. In the same conditions, the all-polymer PM6:PY-IT OPVs demonstrate impressive performance, achieving a maximum PCE of 15% for a 0.09 cm^2^ surface area and 13.68% for a 0.25 cm^2^ surface area. However, for a more accurate comparison of the two systems, it is crucial to analyze the statistical data.

The average value is crucial in evaluating the overall performance of the systems as it provides a more reliable measure than peak values, which may be influenced by outliers or specific conditions. When considering the average values reported in Table 3 across multiple measurements, the difference in efficiency becomes less pronounced (Figure 6b). While the PM6:Y12-based devices exhibit an overall higher peak performance, the average efficiencies across different cells are relatively similar between PM6:Y12 and PM6:PY-IT blends, especially for the 0.25 cm^2^ cells. The losses observed when transitioning from the smallest to the largest surface areas are similar for both systems, with a reduction of 11.4% for PM6:Y12 and 12.1% for PM6:PY-IT. This suggests that PM6:PY-IT exhibits nearly the same potential for high performance when processed using blade-coating from a nonhalogenated solvent system. Figure S14 shows the reproducibility of *V*_oc_, *J*_sc_, and FF for photovoltaic devices based on PM6:Y12 and PM6:PY-IT blends as a function of the surface area. While *V*_oc_ remains consistently stable, *J*_sc_ is the most variable factor among the performance parameters, showing significant fluctuations both between the two systems and within each system as a function of the cell surface area (loss of 7.7% and 6.3% between 0.09 and 0.25 cm^2^ for PM6:Y12 and PM6:PY-IT, respectively). PM6:PY-IT consistently exhibits lower *J*_sc_ values compared to PM6:Y12 with losses rising to 11–13% across both systems, regardless of the surface area. These losses are identical to those of the PCE, showing the direct source of variation. This variation in *J*_sc_ can be attributed to factors such as a less optimized morphology, as evidenced by AFM analysis, and lower charge carrier mobilities determined via SCLC measurements, which collectively result in reduced charge transport and increased recombination. In contrast, FF values remain relatively stable across both systems and for all cell sizes (loss of 2.5–3.9%), suggesting that the factors governing charge extraction and device efficiency at the interfaces are less sensitive to materials and surface area variations.

However, the FF is the most significantly affected parameter when the PM6:PY-IT ink is not subjected to the vortex mixing step. Figure S15 shows the reproducibility of PCE, *V*_oc_, *J*_sc_, and FF for photovoltaic devices based on PM6:PY-IT inks not subjected to the vortex step as a function of the surface area. The average values are reported in Table S2. Notably, a loss of up to 10% is observed for PM6:PY-IT cells with an area of 0.25 cm^2^ when the vortex step is not applied. This observation can be directly correlated to the less well-formed face-on molecular packing and less suitable crystallinity observed by GIWAXS on the corresponding layers as observed in Figure S10b.

Figure 7 demonstrates the EQE spectra of the devices. The integrated *J*_SC_ values are 23.09 mA cm^–2^ and 21.25 mA cm^–2^ for the PM6:Y12 and PM6:PY-IT-based devices, respectively. All measurements were performed on devices with an active area of 0.25 cm^2^. Both PM6:Y12- and PM6:PY-IT-based devices exhibit a broad photon response across the 350–850 nm wavelength range, with maximum EQE values exceeding 75%, highlighting their excellent photoelectric conversion efficiencies. This is further validated by the emergence of two distinct peaks, demonstrating that both the donor (PM6) and acceptor (Y12 or PY-IT) actively participate in the conversion process. Consequently, the well-defined background structural organization observed in the AFM images plays a crucial role in enhancing the PCE by effectively minimizing the impact of aggregates on all-polymer PM6:PY-IT blends.

**Figure 7:**
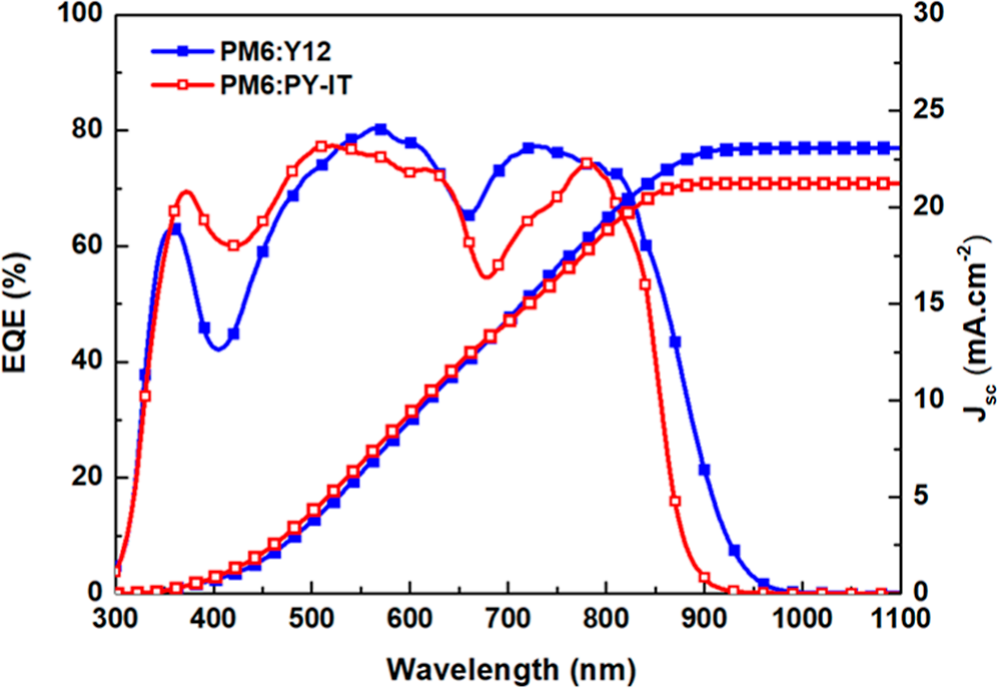
EQE spectra and integrated *J*_sc_ of OPVs based on PM6:Y12 and PM6:PY-IT blends.

Notably, the subtle differences observed in the absorption spectra of Figure 1d are also reflected in the EQE spectra, highlighting a direct correlation between the optical absorption and device performance. The slightly higher absorption observed in the 350–550 nm range for PM6:PY-IT blends contributes to the enhancement of the EQE within the corresponding spectral region. On the contrary, the sharper and substructured peak of PY-IT results in a lower EQE in the 700–800 nm range. The integrated *J*_sc_ values are consistent with those obtained from *J*–*V* measurements, falling within the experimental error margin and confirming the reliability of both measurement techniques. The higher *J*_sc_ values of the PM6:Y12-based device can be ascribed to the enhanced molecular aggregation feature in the photoactive blend.

## 4 Conclusion

We have successfully demonstrated the scalable fabrication of all-polymer OPV devices based on the PM6:PY-IT blend, utilizing doctor-blade coating at ambient temperature in air. *O*-Xylene, employed as a more eco-friendly solvent alternative to traditional halogenated solvents as CF, reinforces the environmental sustainability of this approach. In addition, this study offers a comprehensive comparative analysis with PM6:Y12 solar cells as part of the cutting-edge research into OPVs employing a small-molecule acceptor. For both systems, we implemented an innovative fabrication method for solar cells, utilizing BHJs dissolved in *o*-xylene and deposited through air-processed doctor-blade coating. This novel approach combines the benefits of environmentally friendly solvent processing with scalable film deposition techniques, presenting significant potential for cost-effective and sustainable OPV manufacturing. Both blends demonstrated balanced charge mobilities, a critical factor for efficient charge extraction and minimizing recombination losses. This achievement arises from the meticulous optimization of the ink formulation, including the precise tuning of the donor-to-acceptor ratio and the critical processes for dissolving and homogenizing the all-polymer-based solution. Incorporating simple steps, alongside controlled heating and stirring, such as vortex mixing to enhance solubility and blend homogeneity, clearly demonstrates how processing control can directly influence film morphology and device performance, as confirmed by AFM, TEM, and GIWAXS analyses.

While centered on all-polymer solar cells, our findings demonstrate that the efficiencies of PM6:Y12-based OPV devices not only match but also surpass the values reported in the literature for spin-coated blends treated with CF, highlighting a novel and significant advancement in this area. A crucial and highly significant finding of our study is that the average efficiencies across different cells are remarkably similar between the PM6:Y12 and PM6:PY-IT blends, particularly for the 0.25 cm^2^ devices. The all-polymer PM6:PY-IT OPVs exhibited exceptional performance, achieving a maximum PCE of 15% for a 0.09 cm^2^ surface area and 13.68% for a 0.25 cm^2^ surface area. These results suggest that PM6:PY-IT has significant potential to achieve high performance when processed via blade-coating in air using a nonhalogenated solvent system.

The use of solution-processable donor–acceptor blends ensures strong compatibility with roll-to-roll fabrication and printing techniques, making them highly attractive for cost-effective, large-scale production. The demonstrated efficient FF and balanced charge transport further emphasize the potential for integrating these systems into scalable processes, paving the way for reliable, high-throughput OPV fabrication suitable for commercialization.

## Supplementary Material



## Data Availability

The authors confirm that the data supporting the findings of this study are available within the article and its Supporting Information.

## Notes

Literature review on all-polymer PM6:PY-IT-based solar cells, absorption spectra of neat materials (PM6, Y12, PY-IT) and PM6:PY-IT blends, AFM images of neat materials (PM6, Y12, and PY-IT) and PM6:Y12 and PM6:PY-IT blends, TEM images of PM6:PY-IT blends, GIWAXS patterns of neat materials (PM6, Y12, and PY-IT) and PM6:Y12 and PM6:PY-IT blends, transport properties analyzed by SCLC, and solar cell analysis (PDF)
