# Supplementary material for: Blade-Coated All-Polymer Organic Solar Cells with 15% Efficiency Using Eco-Friendly Solvent Systems
Source: ACS Appl Mater Interfaces. 2025 Oct 8;17(42):58479–90. doi: 10.1021/acsami.5c12486 (PMC12557223; doi:10.1021/acsami.5c12486)
Supplement: Supplementary file 1 [file am5c12486_si_001.pdf]

Supporting Information

Blade-coated all-polymer organic solar cells with 15% efficiency using eco-friendly solvent systems

Mohamed el Amine Kramdi [1,2], Aral Karahan [1], Lydia Abbassi [1,2],

Takeshi Watanabe [3], Hidehiro Sekimoto [4], Olivier Margeat [1], Jörg Ackermann [1],

Carmen M. Ruiz Herrero [2,], Christine Videlot-Ackermann [1,*]*

[1] Aix Marseille Univ., CNRS UMR 7325, CINaM, Campus of Luminy, Case 913, 13288 Marseille Cedex 09, France.

[2] Aix Marseille Univ., Univ. de Toulon, UMR CNRS 7334, IM2NP, Technopôle de Château Gombert, 5 rue Enrico Fermi, 13453 Marseille Cedex 13, France

[3] Industrial Application Division, Japan Synchrotron Radiation Research Institute (JASRI), Sayo, Hyogo 679-5198, Japan

[4] Department of Physical Science and Materials Engineering, Iwate University, Ueda Morioka 020 8551, Japan

Corresponding Authors

* E-mail: carmen.ruiz-herrero@im2np.fr (Carmen Maria Ruiz) and christine.videlot-ackermann@cnrs.fr (Christine Videlot-Ackermann)

1- Literature review on the references related to all-polymer PM6:PY-IT based solar cells

Table S1. Conditions and performance parameters for all-polymer PM6:PY-IT based solar cells.

Year	ratio	solvant	Depsotion technic	V_{oc} (V)	J_{sc} (mA.cm^{-2})	FF (%)	PCE (%)	Surface area (cm^2)	Ref
2020	1:1	CF+1vol% CN	SC	0.933	22.3	72.3	15.05	0.059	1
2021	1:1	CF+1vol% CN	SC	0.937	21.9	73.6	15.11	0.059	2
	1:1	CF+1vol% CN	SC	0.932	22.31	71.8	14.93	0.059	3
		CF+0.7vol% CN		0.936	21.41	73.6	14.77		
	/(1)	/(1)	/(1)	0.936	22.77	73.2	15.82	/(1)	4
	1:1	CF+1vol% CN	SC	0.932	22.18	72.5	15	0.059	5
		toluene++1vol% CN		0.933	22.52	73.8	15.51		
	/(1)	/(1)	/(1)	0.947	22.78	74.6	16.1	/(1)	6
2022	1:1	CF+1vol% CN	SC	0.925	23.35	71.05	15.35	0.06	7
	1:1	CF+1vol% CN	SC	0.934	22.6	72.2	15.24	0.059	8
	1:1	o-xy+1vol% CN	SC	0.942	23.39	75.3	16.59	0.04	9
	1:1.2	toluene	SC	0.93	23	70.1	15	0.04	10
	1:1	CF+1vol% CN	MAC	0.945	23.27	70.77	15.53	0.04	11
	1:1	toluene+1vol% CN	SC	0.933	22.62	73.7	15.55	0.059	12
		o-xy+1vol% CN		0.938	23.15	74.7	16.22		
		TMB+1vol% CN		0.944	22.04	73.9	15.38		
	1.2:1	CF	SC	0.92	23.27	70.55	15.1	0.047	13
	1:1	toluene+1vol% CN	SC	0.932	23.17	71.03	15.34	0.04	14
		CF+1vol% CN		0.931	23.22	71.65	15.49		
		toluene+1vol% CN	BC	0.929	21.35	70.26	13.94	1.21	
		CF+1vol% CN		0.925	20.73	63.19	12.12		
	1:1.2	CF+1vol% CN	SC	0.947	22.48	72.8	15.47	0.04	15
	1:1	CF+1vol% CN	SC	0.948	22.31	73.5	15.56	0.059	16
		o-xy		0.931	22.52	61.3	12.86		
	1:1.2	toluene+1vol% CN	SC	0.93	23	70.1	15	0.04	17
				0.93	23.4	59.1	12.9	1	
	1:1	o-xy+1vol% CN	SC	0.945	23.57	76.9	17.14	0.059	18
	1:1	CF+CN	SC	0.94	22.96	71.77	15.49	0.04	19
	1:1	CF,CB	LBL,SC	0.95	22.61	73.62	15.81	0.038	20
		CF	SC	0.95	22.29	72.24	15.29		
	/(1)	CF+1vol% CN	SC	0.928	23.1	69.22	14.84	/(1)	21
2023	1:1.2	CF+2vol% CN	SC	0.944	24.4	75.48	17.38	0.06	22
	1:1.2	CF+1.5vol% CN	SC	0.935	23.56	73.69	16.27	0.0936	23
	1:1	o-xy+1vol% CN	SC	0.942	23.39	75.3	16.59	0.04	24
	1:1	CF+CN	SC	0.936	22.8	72.12	15.39	0.1	25
	1:1	CF+1vol% CN	SC	0.937	22.93	71.09	15.28	0.1	26
	1:1.2	CF+2vol% CN	SC	0.941	23.4	75.7	16.7	0.04	27
	1:1	CF+1vol% CN	SC	0.921	23.21	71.1	15.2	0.03	28
	1:1.1	CF+1.5vol% CN	SC	0.943	24.13	70.72	16.09	0.06	29
	/(1)	CF,CB	LBL	0.95	23.29	72.51	16.04	0.038	30
	1:1.2	CF	SC	0.945	26.37	76.48	19.06	/(1)	31
	/(1)	/(1)	SC	0.931	23.92	74.28	16.65	0.04	32
2024	1:1.2	CF+1vol% CN	SC	0.936	22.76	70.79	15.08	/(1)	33
	1:1.2	CF+2vol% CN	SC	0.91	23.27	70.2	14.8	0.04	34
2025	1:1	CF	SC	0.953	24.48	63.79	14.71	0.054	35
		CF+2vol% MN		0.938	25.27	70.82	16.79		
	1:1	o-xy+2vol% CN	SC	0.937	23.17	73.06	15.84	0.045	36
	1:1.2	CF	SC	0.93	22.15	63.57	13.11	/(1)	37
	1:1.2	CF+1.5vol% CN	SC	0.93	23.35	71.38	15.53	0.057	38
				0.94	24.6	72.25	16.69		

With SC = spin-coating, LBL = Layer-by-layer, BC = blade-coating.

(1) no indication in the reference

Figure S1 gives a graphical representation of the distribution of PCEs listed in **Table S1** over the years, with additionnaly the values obtained in this work highlighted in red. A differentiation is made for the values obtained by SC and DB. It is worth noting that the overwhelming majority of PCEs of **Tables S1** (in solid black circles in the **Figure S1**) are obtained for solar cells with surface areas not extended 0.06 cm^2. In our study, surface areas of 0.09 cm^2 and 0.25 cm^2 were utilized.

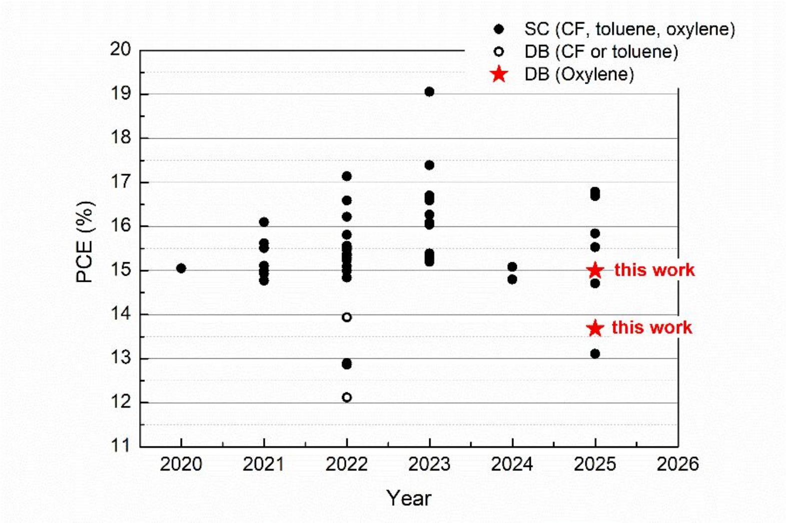

Figure S1. Graphical representation of the distribution of PCEs listed in **Table S1** over the years, with the values obtained in this work highlighted in red. A differentiation is made for the values obtained by SC and DB.

As demonstrated by the **Table S1**, the most commonly used solvents are chloroform (CF) and toluene. Chloroform is highly effective at dissolving a wide range of polymers and moderately polar organic compounds, but its high volatility and tendency to degrade into toxic byproducts under light and oxygen exposure make it suitable mainly for use in inert atmospheres. By consequence, it is primarily used for deposition processes under controlled atmospheres. In contrast, toluene is less volatile and less toxic, providing a more air-stable option while maintaining good solubility for non-polar materials, particularly those used in organic photovoltaic devices. However, toluene has a tendency to absorb moisture from the air, which can lead to inconsistent film formation and reproducibility issues during deposition in ambient conditions.

Therefore, when aiming for consistent results in ambient conditions, *o*-xylene is generally the preferred solvent, as it provides a very good alternative for deposition processes in air with lower toxicity [39]. O-xylene, with its higher boiling point and low volatility, enables slower drying, which is advantageous for forming thicker, more uniform films [40]. O-xylene has also a low moisture uptake and offers greater stability during air processing. It is especially well-suited for applications that require precise control over film morphology, such as in high-performance organic semiconductors [41]. Consequently, *o*-xylene emerges as the most stable choice for air-based deposition [42], even though it has been utilized by SC in controlled storage conditions in

reférences of **Tables S1**. Therefore, in the present study, we have chosen to fully explore the prerequisites of this solvent for air-based deposition studies by DB.

The graph in **Figure S2**, which values are taken from **Table S1**, shows that the most studied ratio is the 1:1 ratio compared to the 1:1.2 ratio. Furthermore, our studies on hole and electron mobility determination, carried out through SCLC, demonstrate higher mobilities for the 1:1 ratio with a better balance of values (see **Figure S12**).

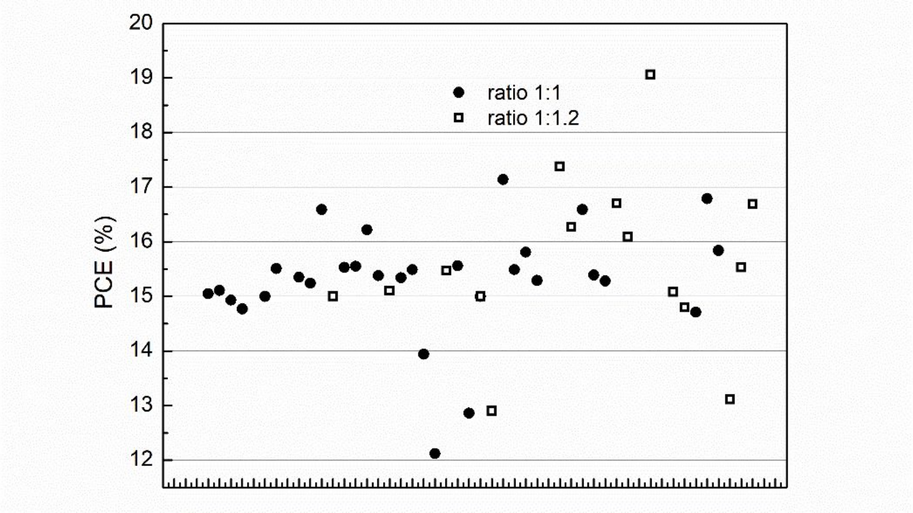

Figure S2. Graphical representation of the distribution of PCEs listed in **Table S1** as function of the ratio PM6:PY-IT.

2- Absorption spectra

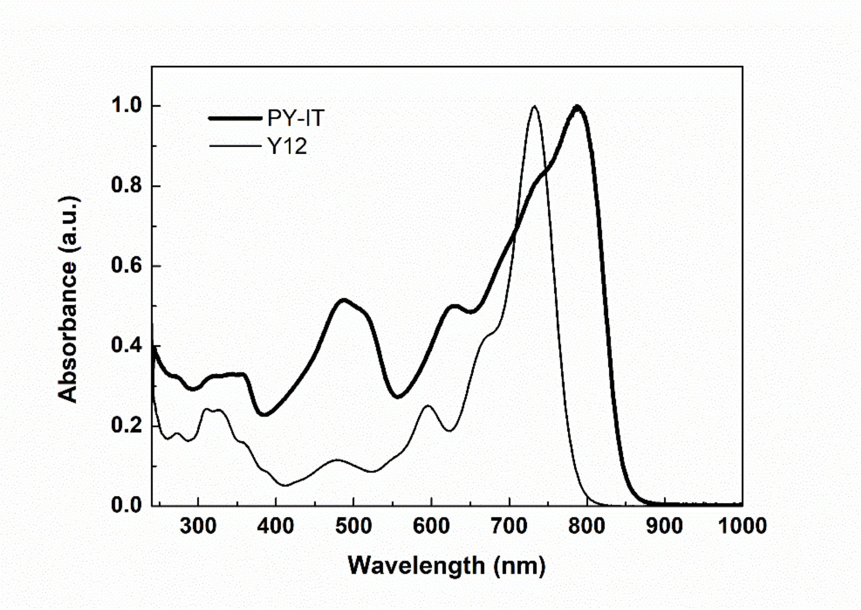

Figure S3. Normalized absorption spectra of Y12 and PY-IT in chloroform (CF) solution.

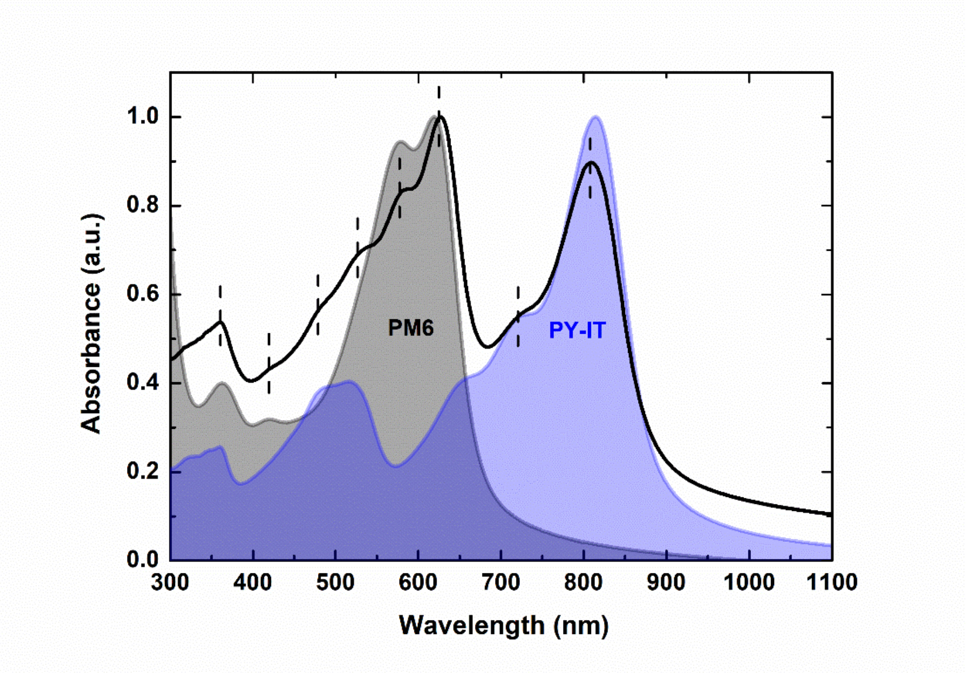

Figure S4. Normalized absorption spectra of PM6 (grey), PY-IT (blue) and PM6:PY-IT (black line) thin films. Dashed lines are used as visual guides to indicate the peak positions corresponding to the material structures.

3- Surface and bulk analysis

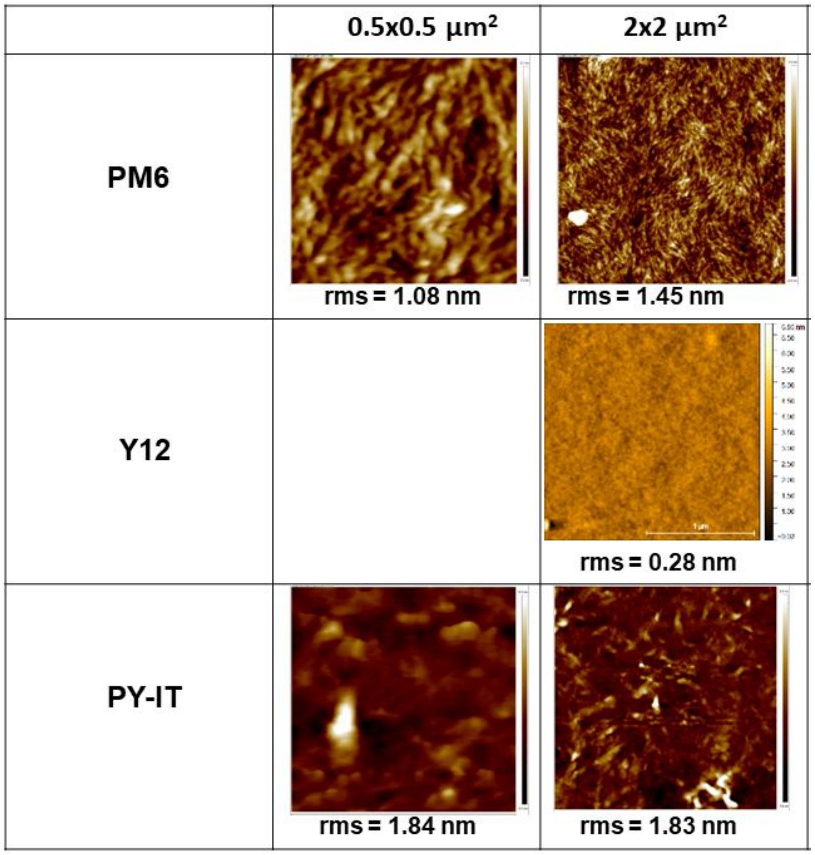

Figure S5. AFM images of neat materials (PM6, Y12 and PY-IT). Inks subjected to the vortexing procedure

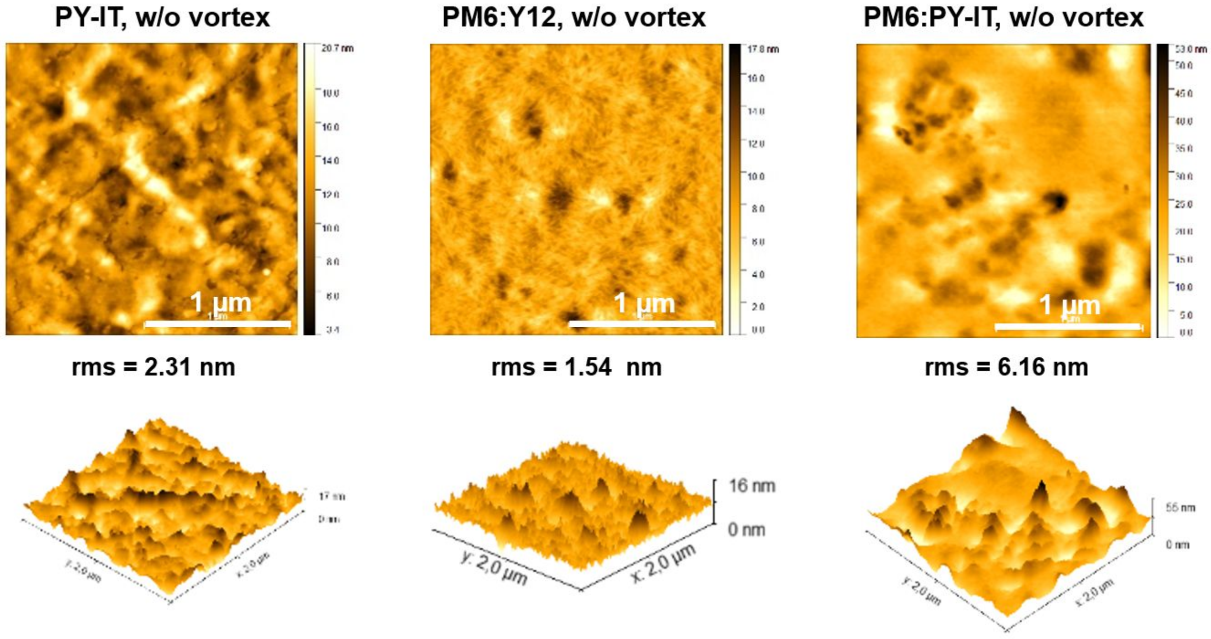

Figure S6. AFM images of PY-IT, PM6:Y12 and PM6:PY-IT. Inks not subjected to the vortexing procedure.

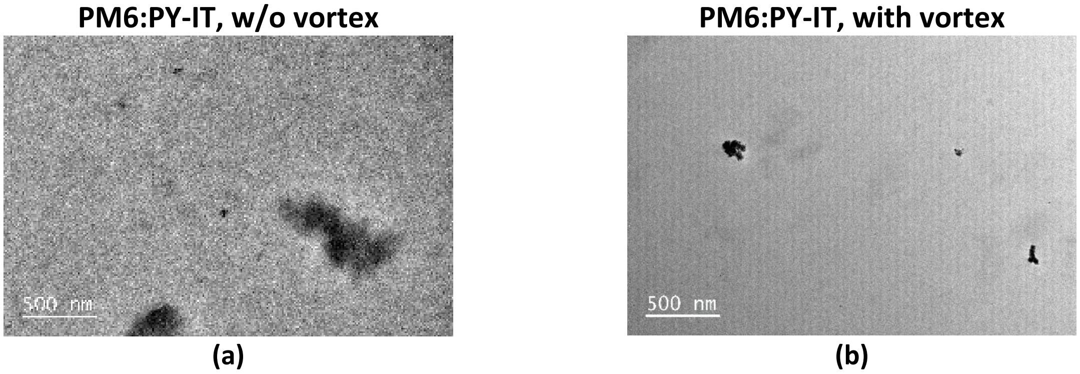

Figure S7. TEM images of PM6:PY-IT film with inks not subjected (a) or subjected (b) to the vortexing procedure.

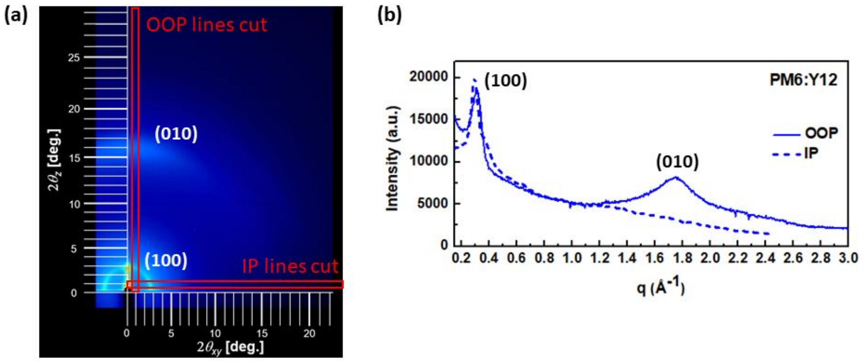

Figure S8. (a) 2D-grazing incidence X-ray diffraction pattern with indication of the most prominent reflexion and (b) corresponding out-of-plane (OOP) and in-plane (IP) profiles of PM6:Y12. Figure 4a shows the raw data observed by the 2D detector, while Figure S8a is the corrected data to accurately represent q. To obtain the OOP and IP profiles, lines cut were selected in the red areas of the pattern and the conversion to q profiles was performed using $q = (4\pi/\lambda) \sin\theta$ with θ in radians.

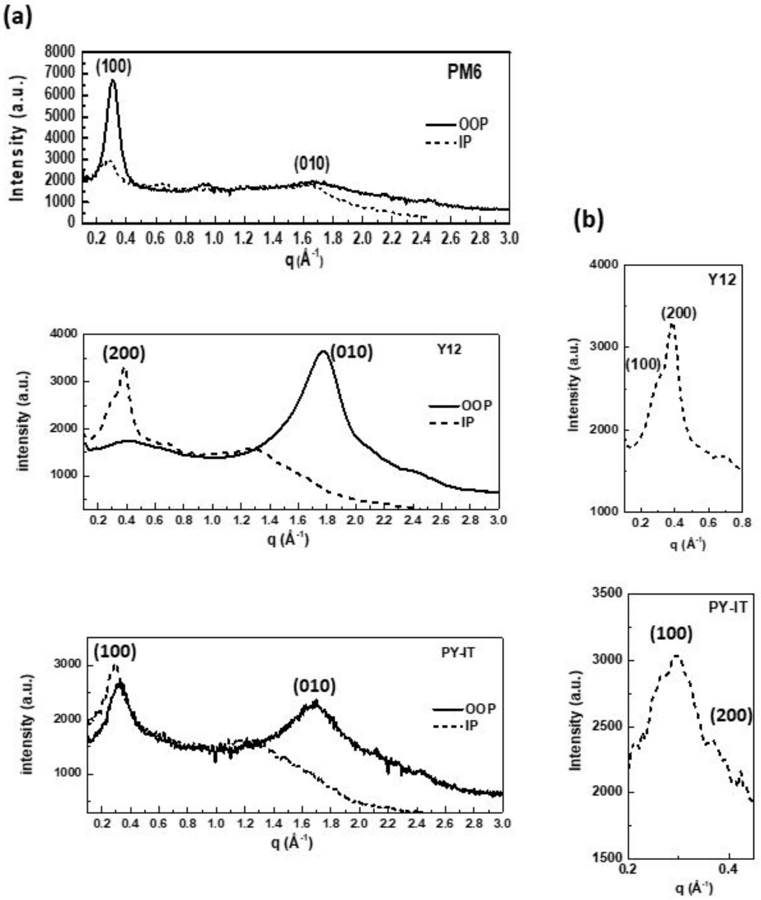

Figure S9. (a) Out-of-plane (OOP) and in-plane (IP) profiles of neat materials (PM6, Y12 and PY-IT). (b) Zoomed-in view of the IP profiles.

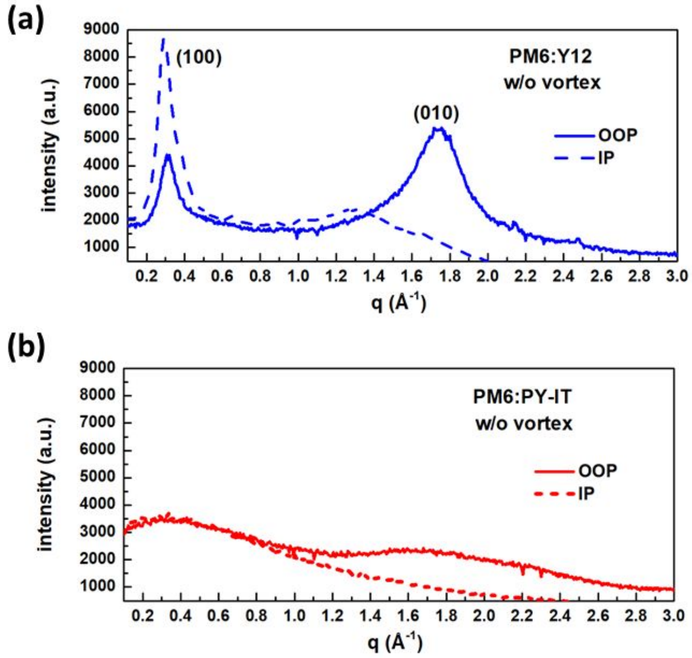

Figure S10. Out-of-plane (OOP) and in-plane (IP) profiles of PM6:Y12 (a) and PM6:PY-IT (b) blends, with both inks not subjected to the vortexing procedure.

4- Transport properties by SCLC

4.1 Hole only devices based on PM6:PY-T (raio 1:1)

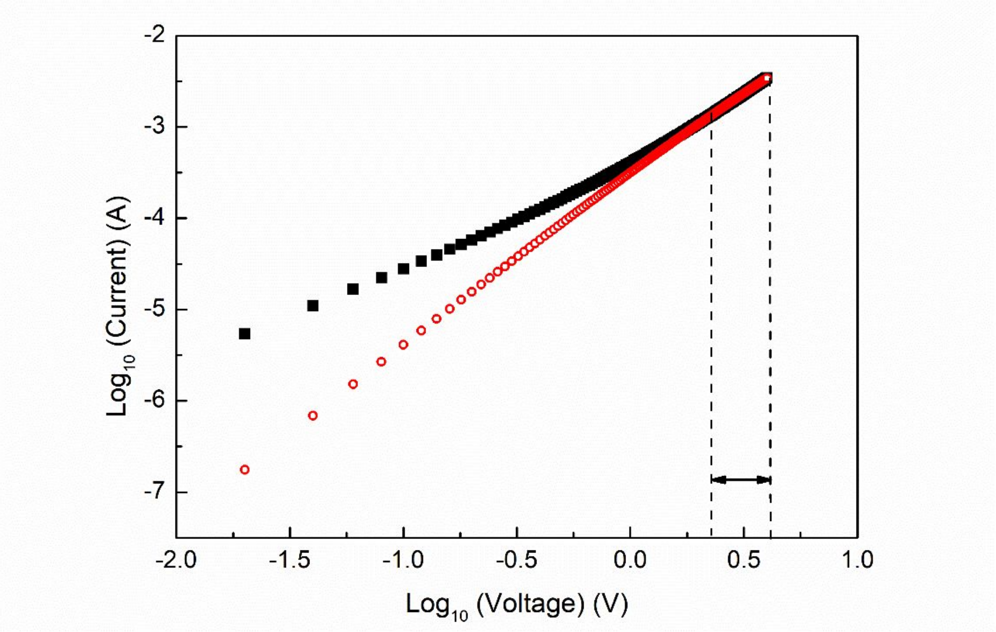

Figure S11. Plot on a double logarithmic scale of I-V curve (black square) together with the best fit (red circle) against the logarithm of the I-V curve by equation S1 for a hole-only device.

Data fitting by SCLC protocol for Figure S8:

To fit against the logarithm of the I-V curve, the fitting equation in base-10 logarithm used is:

$$Log_{10}(I) = Log_{10}\left(A\mu_0 \frac{9}{8}\frac{\varepsilon\varepsilon_0}{d^3}\right) + 2Log_{10}(V_{int}) + \frac{0.387\gamma}{\sqrt{d}} 10^{Log_{10}(V_{int})/2}$$

Equation S1

Data fitting reporting:

Fitted voltage range: 2.3-4 V (represented in the Figure S11 by the vertical dotted lines)

Fixed parameters:

$d = 1.38\times10^{-5}$ cm

$A = 0.09$ cm^2

$\varepsilon = 3.5$

$\varepsilon_0 = 8.85 \times 10^{-14}$ F.cm^{-1}

Fitted Parameters:

$\mu_0 = 3.89\times10^{-4}$ cm^2/Vs

$\gamma = -1.2\times10^{-5}$ cm$^{1/2}$/V$^{1/2}$

Good fit over voltage range: 2.3-4 V

Corresponding field range: 1.67-2.9×10^5 V/cm

Mobility at defined field with: $\mu_h = \boldsymbol{\mu_0}.\exp(\boldsymbol{\gamma}.\sqrt{F})$ is $\mu = 3.78\times10^{-4}$ cm^2/Vs at $F = 2\times10^5$ V/cm.

4.2 Electron only devices based on PM6:PY-T (raio 1:1)

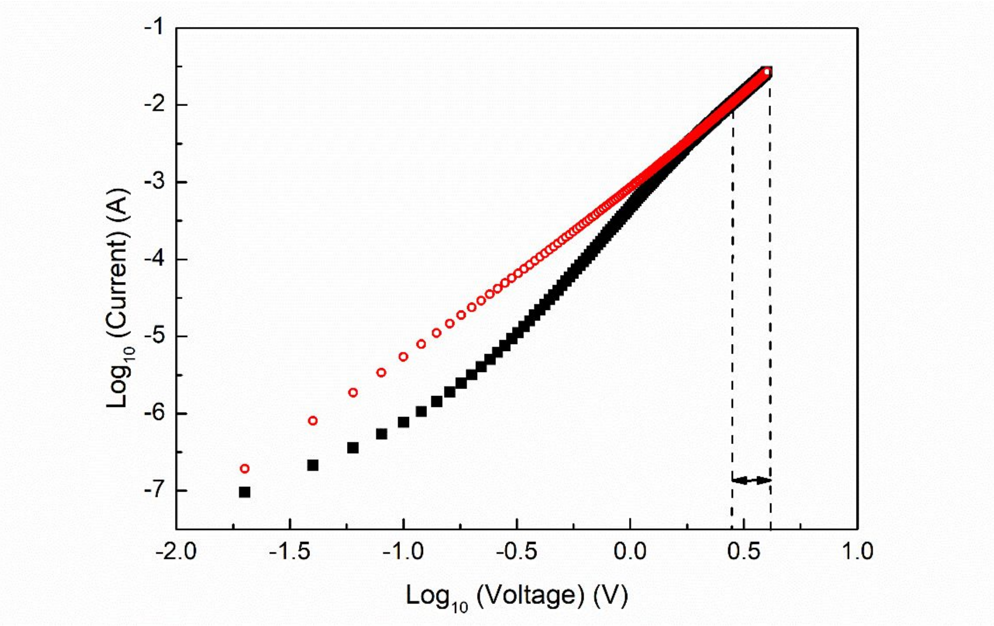

Figure S12. Plot on a double logarithmic scale of I-V curve (black square) together with the best fit (red circle) against the logarithm of the I-V curve by equation S1 for an electron-only device.

Data fitting by SCLC protocol for Figure S9:

To fit against the logarithm of the I-V curve, the fitting equation in base-10 logarithm used is:

$$Log_{10}(I) = Log_{10}\left(A\mu_0 \frac{9}{8}\frac{\varepsilon\varepsilon_0}{d^3}\right) + 2Log_{10}(V_{int}) + \frac{0.387\gamma}{\sqrt{d}}10^{Log_{10}(V_{int})/2}$$

Equation S1

Data fitting reporting:

Fitted voltage range: 3-4 V (represented in the Figure S12 by the vertical dotted lines)

Fixed parameters:

$d = 1.16\times10^{-5}$ cm

$A = 0.09$ cm^2

$\varepsilon = 3.5$

$\varepsilon_0 = 8.85 \times 10^{-14}$ F.cm^{-1}

Fitted Parameters:

$\mu_0 = 2.18\times10^{-5}$ cm^2/Vs

$\gamma = 2.56\times10^{-3}$ cm$^{1/2}$/V$^{1/2}$

Good fit over voltage range: 3-4 V

Corresponding field range: 2.59-3.45×10^5 V/cm

Mobility at defined field with: $\mu_h = \boldsymbol{\mu_0}.\exp(\boldsymbol{\gamma}.\sqrt{F})$ is $\mu = 8.89\times10^{-5}$ cm^2/Vs at $F = 3\times10^5$ V/cm.

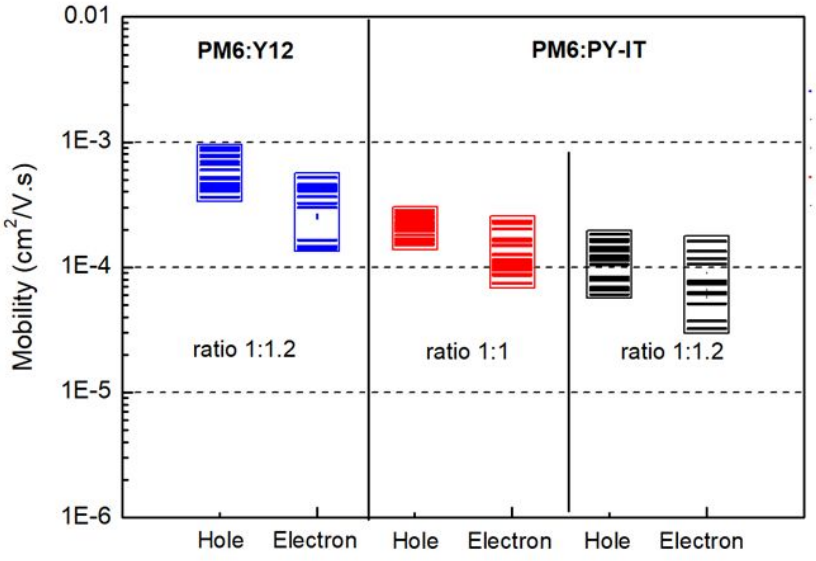

Figure S13. Comparative distribution of hole and electron mobilities as a function of blend layer compositions: PM6:Y12 (ratio 1:1.2), PM6:PY-IT (ratio 1:1) and PM6:PY-IT (ratio 1:1.2).

5- Solar cell analysis

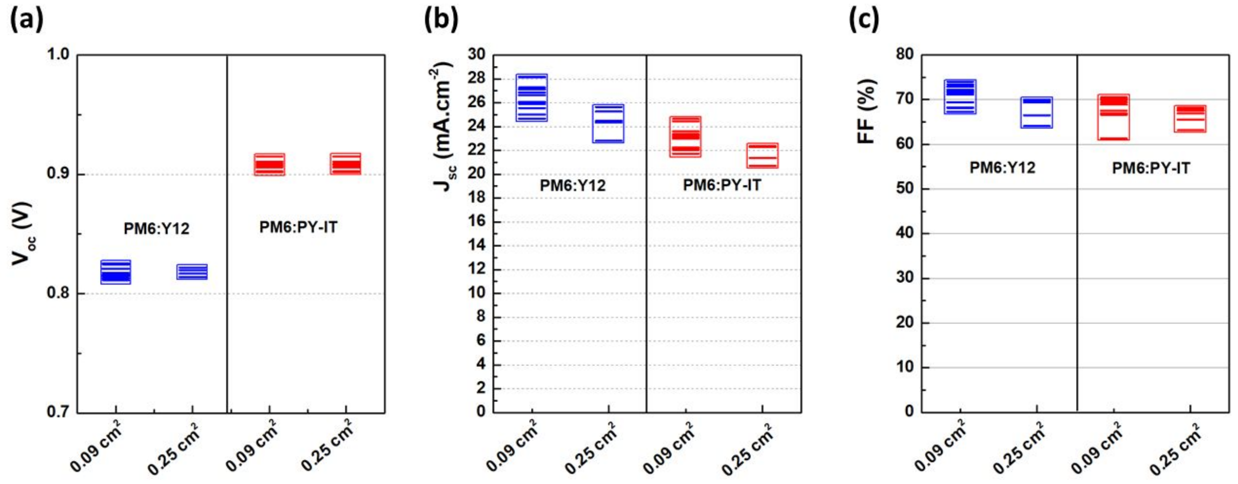

Figure S14. V_{oc} (a), J_{sc} (b) and FF (c) distributions as function of cell area. The ink formulation was prepared with the vortex mixing step.

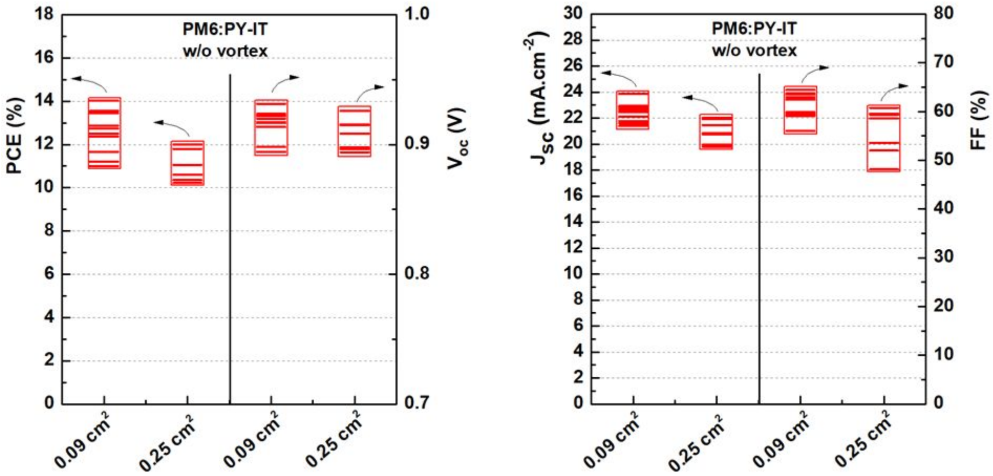

Figure S15. PCE, V_{oc}, J_{sc} and FF distribution as function of cell area. The ink formulation was prepared without the vortex mixing step.

Table S2. Photovoltaic parameters of PM6:Y12 and PM6:PY-IT blends as function of cell area. The ink formulation was prepared without the vortex mixing step.

	Aera (cm^2)	V_{oc} (V)	J_{sc} (mA/cm^2)	FF (%)	PCE (%)
PM6:PY-IT	0.09	0.922<br>(0.915±0.011)	28.85<br>(22.43±0.74)	63.73<br>(61.09±2.72)	14.02[a]<br>(12.53±1.01)
	0.25	0.896<br>(0.907±0.011)	22.03<br>(20.96±0.89)	60.77<br>(56.00±4.78)	12.00[a]<br>(11.00±0.75)

[a] Values in this line are parameters associated with the devices that showed the highest PCEs.

References

[1] Zhenghui Luo, Tao Liu, Ruijie Ma, Yiqun Xiao, Lingling Zhan, Guangye Zhang, Huiliang Sun, Fan Ni, Gaoda Chai, Junwei Wang, Cheng Zhong, Yang Zou, Xugang Guo, Xinhui Lu, Hongzheng Chen, He Yan, Chuluo Yang. Adv. Mater. 2020, 32, 2005942.

[2] Tao Liu, Tao Yang, Ruijie Ma, Lingling Zhan, Zhenghui Luo, Guangye Zhang, Yuan Li, Ke Gao, Yiqun Xiao, Jianwei Yu, Xinhui Zou, Huiliang Sun, Maojie Zhang, Top Archie Dela Peña, Zengshan Xing, Heng Liu, Xiaojun Li, Gang Li, Jianhua Huang, Chunhui Duan, Kam Sing Wong, Xinhui Lu, Xugang Guo, Feng Gao, Hongzheng Chen, Fei Huang, Yongfang Li, Yuliang Li, Yong Cao, Bo Tang, He Yan. Joule, 2021, 5, 914-930.

[3] Ruijie Ma, Jianwei Yu, Tao Liu, Guangye Zhang, Yiqun Xiao, Zhenghui Luo, Gaoda Chai, Yuzhong Chen, Qunping Fan, Wenyan Su, Gang Li, Ergang Wang, Xinhui Lu, Feng Gao, Bo Tang, He Yan. Aggregate, 2022, 3:e58.

[4] Ruijie Ma, Miao Zeng, Yixin Li, Tao Liu, Zhenghui Luo, Ye Xu, Ping Li, Nan Zheng, Jianfeng Li, Yuan Li, Runfeng Chen, Jianhui Hou, Fei Huang, He Yan. Adv. Energy Mater., 2021, 11, 2100492.

[5] Le Jin, Ruijie Ma, Heng Liu, Wenhan Xu, Zhenghui Luo, Tao Liu, Wenyan Su, Yuxiang Li, Rui Lu, Xinhui Lu, He Yan, Ben Zhong Tang, Tao Yang. ACS Appl. Mater. Interfaces 2021, 13, 34301-34307.

[6] Siyi Ding, Ruijie Ma, Tao Yang, Guangye Zhang, Junli Yin, Zhenghui Luo, Kai Chen, Zongcheng Miao, Tao Liu, He Yan, Dongfeng Xue. ACS Appl. Mater. Interfaces, 2021, 13, 51078-51085.

[7] Ke Hu, Jiaqi Du, Can Zhu, Wenbin Lai, Jing Li, Jingming Xin, Wei Ma, Zhanjun Zhang, Jinyuan Zhang, Lei Meng, Yongfang Li. Sci China Chem, 2022, 65, 954-963.

[8] Shangfei Yao, Tao Yang, Xiaodong Shen, Tongzhou Li, Bingzhang Huang, Heng Liu, Xinhui Lu, Tao Liu, Bingsuo Zou. J. Mater. Chem. C, 2022, 10, 9723-9729.

[9] Tao Liu, Kangkang Zhou, Ruijie Ma, Libin Zhang, Ciyuan Huang, Zhenghui Luo, Hongxiang Zhu, Shangfei Yao, Chuluo Yang, Bingsuo Zou, Long Ye. Aggregate, 2023; 4:e308.

[10] Kaihu Xian, Kangkang Zhou, Mingfei Li, Junwei Liu, Yaowen Zhang, Tao Zhang, Yong Cui, Wenchao Zhao, Chunming Yang, Jianhui Hou, Yanhou Geng, and Long Ye. Chin. J. Chem., 2023, 41, 159-166.

[11] Yuchen Yue, Bing Zheng, Wenjie Yang, Lijun Huo, Jingxia Wang, Lei Jiang. Adv. Mater., 2022, 34, 2108508.

[12] Tao Yang, Shangfei Yao, Tao Liu, Bingzhang Huang, Yiqun Xiao, Heng Liu, Xinhui Lu, Bingsuo Zou. ACS Appl. Mater. Interfaces, 2022, 14, 29956-29963.

[13] Wenqing Zhang, Chenkai Sun, Indunil Angunawela, Lei Meng, Shucheng Qin, Liuyang Zhou, Shaman Li, Hongmei Zhuo, Guang Yang, Zhi-Guo Zhang, Harald Ade, Yongfang Li. Adv. Mater., 2022, 34, 2108749.

[14] Jiabin Liu, Jiawei Deng, Yangyang Zhu, Xiaokang Geng, Lifu Zhang, Sang Young Jeong, Dan Zhou, Han Young Woo, Dong Chen, Feiyan Wu, Lie Chen. Adv. Mater., 2023, 35, 2208008.

[15] Yanna Sun, Ruijie Ma, Yuanyuan Kan, Tao Liu, Kangkang Zhou, Pengke Liu, Jin Fang, Yiyao Chen, Long Ye, Changqi Ma, He Yan, Ke Gao. Macromol. Rapid Commun., 2022, 43, 2200139.

[16] Ruijie Ma, Kangkang Zhou, Yanna Sun, Tao Liu, Yuanyuan Kan, Yiqun Xiao, Top Archie Dela Peña, Yixin Li, Xinhui Zou, Zengshan Xing, Zhenghui Luo, Kam Sing Wong, Xinhui Lu, Long Ye, He Yan, Ke Gao. Matter, 2022, 5, 725-734,.

[17] Kaihu Xian, Kangkang Zhou, Mingfei Li, Junwei Liu, Yaowen Zhang, Tao Zhang, Yong Cui, Wenchao Zhao, Chunming Yang, Jianhui Hou, Yanhou Geng, Long Ye. Chin. J. Chem., 2023, 41, 159-166.

[18] Shasha Shi, Yiwen Hou, Tao Yang, Ciyuan Huang, Shangfei Yao, Chenfu Zhao, Yudie Liu, Ziyang Zhang, Tao Liu, Bingsuo Zou. ACS Omega, 2022, 7, 41789-41795.

[19] Yuchen Yue, Bing Zheng, Jianling Ni, Wenjie Yang, Lijun Huo, Jingxia Wang, Lei Jian. Adv. Sci., 2022, 9, 2204030.

[20] Wenjing Xu Xixiang Zhu, Xiaoling Ma, Hang Zhou, Xiong Li, Sang Young Jeong, Han Young Woo, Zhengji Zhou, Qianqian Sun, Fujun Zhang. J. Mater. Chem. A, 2022, 10, 13492-13499.

[21] Xin Yu, Hui Lin, Minglang Li, Benteng Ma, Ruixuan Zhang, Xiaoyang Du, Caijun Zheng, Gang Yang, Silu Tao. Organic Electronics, 2022, 104, 106471

[22] Tianyi Chen, Xiangjun Zheng, Di Wang, Yuxuan Zhu, Yanni Ouyang, Jingwei Xue, Mengting Wang, Shanlu Wang, Wei Ma, Chunfeng Zhang, Zaifei Ma, Shuixing Li, Lijian Zuo, Hongzheng Chen. Adv. Mater., 2024, 36, 2308061.

[23] Jianxiao Wang, Chenyu Han, Fuzhen Bi, Zunyuan Hu, Shuguang Wen, Yonghai Li, Chunming Yang, Xichang Bao, Junhao Chu. Energy Environ. Sci., 2023, 16, 2327-2337.

[24] Kangkang Zhou, Kaihu Xian, Ruijie Ma, Junwei Liu, Mengyuan Gao, Saimeng Li, Tao Liu, Yu Chen, Yanhou Geng, Long Ye. Energy Environ. Sci., 2023, 16, 5052-5064.

[25] Chenyu Shang, Shuai Zhang, Dong Han, Xiqiang Ding, Yaowen Zhang, Chunming Yang, Jianxu Ding, Xichang Bao. ACS Appl. Mater. Interfaces 2023, 15, 5538-5546.

[26] Shuai Zhang, Mian Cai, Chenyu Shang, Fuzhen Bi, Fan Feng, Zhengkun Du, Cheng Sun, Yonghai Li, Xichang Bao. Adv. Funct. Mater., 2023, 33, 2301701.

[27] Lijiao Ma, Yong Cui, Jianqi Zhang, Kaihu Xian, Zhihao Chen, Kangkang Zhou, Tao Zhang, Wenxuan Wang, Huifeng Yao, Shaoqing Zhang, Xiaotao Hao, Long Ye, Jianhui Hou. Adv. Mater., 2023, 35, 2208926.

[28] Rong Hu, Zijie Xiao, Yurong Liu, Yongyao Su, Chaozhong Guo, Zhe Chen, Cuihong Liu, Wei Zhang. ACS Appl. Mater. Interfaces, 2023, 15, 28321-28331.

[29] Jing Guo, Xinxin Xia, Beibei Qiu, Jinyuan Zhang, Shucheng Qin, Xiaojun Li, Wenbin Lai, Xinhui Lu, Lei Meng, Zhanjun Zhang, Yongfang Li. Adv. Mater., 2023, 35, 2211296.

[30] Wenjing Xu, Miao Zhang, Xiaoling Ma, Xixiang Zhu, Sang Young Jeong, Han Young Woo, Jian Zhang, Wenna Du, Jian Wang, Xinfeng Liu, Fujun Zhang. Adv. Funct. Mater., 2023, 33, 2215204.

[31] Rui Zeng, Lei Zhu, Ming Zhang, Wenkai Zhong, Guanqing Zhou, Jiaxing Zhuang, Tianyu Hao, Zichun Zhou, Libo Zhou, Nicolai Hartmann, Xiaonan Xue, Hao Jing, Fei Han, Yiming Bai, Hongbo Wu, Zheng Tang, Yecheng Zou, Haiming Zhu, Chun-Chao Chen, Yongming Zhang, Feng Liu. Nature Communications, 2023, 14:4148.

[32] Zhixiang Li, Zhe Zhang, Hongbin Chen, Yunxin Zhang, Yuan-Qiu-Qiang Yi, Ziqi Liang, Bin Zhao, Miaomiao Li, Chenxi Li, Zhaoyang Yao, Xiangjian Wan, Bin Kan, Yongsheng Chen. Adv. Energy Mater., 2023, 13, 2300301.

[33] Kaige Ma, Jianghao Jin, Wenfei Shen, Sui Mao, Laurence A. Belfiore, Jianguo Tang. Optical Materials, 2024, 147, 114735.

[34] Kangkang Zhou, Dexia Han, Kaihu Xian, Saimeng Li, Mengyuan Gao, Kai Zhang, Bin Zhao, Xin Li, Yu Chen, Yanhou Geng, Long Ye. Energy Environ. Sci., 2024, 17, 5950-5961.

[35] Luzhuo Li, Hanyue Gao, Mingyu Zuo, Yu Shen, Qiang Zhang, Yanchun Han. J. Mater. Chem. C, 2025, 13, 4983-4992.

[36] Suxiang Ma, Henan Li, Wenchang Wu, Sergio Gámez-Valenzuela, Ruijie Ma, Qingqing Bai, Jianbin Zhong, Sang Young Jeong, Qian Liu, Hao Zhang, Guangye Zhang, Wei Zhang, Junwu Chen, Enmin Huang, Bin Liu, Kui Feng, Han Young Woo, Li Niu, Huiliang Sun, Xugang Guo. Angew. Chem., 2025, 137, e202423616.

[37] Hongli Wang, Hongming Kou, Jiye Pan, Xunchang Wang, Deyu Liu, Renqiang Yang. Small, 2025, 21, 2412767.

[38] Bin Zhang, Zhenshen Pan, Wenming Li, Yushou Zhao, Xiaolan Qin, Aiqin Li, Menglan Lv, Xiaofeng Qin, Weile Guo, Zhicai He, Ergang Wang. Adv. Energy Mater., 2025, 2404297.

[39] Shaoqing Zhang, Long Ye, Hao Zhang, Jianhui Hou. Materials Today, 2016,19, 533-543.

[40] Kang An, Wenkai Zhong, Lei Ying, Peng Zhu, Baobing Fan, Zhenye Li, Ning Li, Fei Huang, Yong Cao. J. Mater. Chem. C, 2020, 8, 270-275.

[41] Yunpeng Qin, Haoran Tu, Nathan Woodward, Mihirsinh Chauhan, Gaurab J. Thapa, Aram Amassian, Justin Neuf, Wei You, Haipeng Yin, Harald Ade. Adv. Energy Sustainability Res., 2025, 2400268.

[42] Peiyao Xue, Pei Cheng, Ray P. S. Han, Xiaowei Zhan. Mater. Horiz., 2022, 9, 194-219.
